# Molecular evidence of RNA polymerase II gene reveals the origin of worldwide cultivated barley

**DOI:** 10.1038/srep36122

**Published:** 2016-10-27

**Authors:** Yonggang Wang, Xifeng Ren, Dongfa Sun, Genlou Sun

**Affiliations:** 1 College of Plant Science and Technology, Huazhong Agricultural University, Wuhan 430070, China; 2 Hubei Collaborative Innovation Center for Grain Industry, Jingzhou, 434025, Hubei, China; 3 Department of Biology, Saint Mary’s University, Halifax, Nova Scotia B3H 3C3, Canada

## Abstract

The origin and domestication of cultivated barley have long been under debate. A population-based resequencing and phylogenetic analysis of the single copy of *RPB2* gene was used to address barley domestication, to explore genetic differentiation of barley populations on the worldwide scale, and to understand gene-pool exchanges during the spread and subsequent development of barley cultivation. Our results revealed significant genetic differentiation among three geographically distinct wild barley populations. Differences in haplotype composition among populations from different geographical regions revealed that modern cultivated barley originated from two major wild barley populations: one from the Near East Fertile Crescent and the other from the Tibetan Plateau, supporting polyphyletic origin of cultivated barley. The results of haplotype frequencies supported multiple domestications coupled with widespread introgression events that generated genetic admixture between divergent barley gene pools. Our results not only provide important insight into the domestication and evolution of cultivated barley, but also enhance our understanding of introgression and distinct selection pressures in different environments on shaping the genetic diversity of worldwide barley populations, thus further facilitating the effective use of the wild barley germplasm.

Barley is one of the oldest, most widely distributed, extensively cultivated, and economically important crops. Cultivated barley (*Hordeum vulgare* L.) is the domesticated descendants of wild barley (*Hordeum spontaneum* L.)^1^. Domestication is the outcome of a selection process that led to increased adaptation to cultivation or rearing and utilization by humans^2^. Previous studies have demonstrated much less variation in cultivated barley in relative to wild barley^3^,^4^,^5^,^6^,^7^, suggesting that cultivated barley originated from small initial wild populations^8^. However, sites of barley domestication events remain under debate. The oldest archaeological remains of barley grains were found at various human Neolithic sites in the Fertile Crescent and traced back to around 8500 calibrated years (cal.) B.C.^1^,^9^,^10^,^11^. The wider distribution wild barley in the Near East Fertile Crescent as well as historic and molecular studies commonly supported that the Near East Fertile Crescent is both a major original center of wild barley and a domestication center of its cultivated form^3^,^9^,^12^,^13^,^14^.

However, since the multiple domestication centers of barley were postulated as early as 1926^15^, the original center of barley cultivation has been widely debated. In addition to the primary habitats of the Fertile Crescent, the natural distribution of *H. spontaneum* in several geographically distinct locations has challenged the prevalent monocentric theory of the origin of barley^16^,^17^. Multiple domestications imply independent origins of many agronomically important mutations^18^,^19^. Studies on row-type of barley demonstrated that six-rowed phenotype originated repeatedly, at different times and in different regions independently, through mutations of *Vrs1*^20^. Distinct genetic loci determining traits-brittle rachis were found in Eastern and Western barleys^18^,^21^. The concept of polyphyletic domestication of cultivated barley was also bolstered by numerous genetic studies^22^,^23^,^24^,^25^. Recent resequencing data from multiple loci, for instance, proposed that barley has been domesticated at least twice in two locations, within the Fertile Crescent and at location 1,500–3,000 km farther East^22^.

The role of wild barley from the Tibetan Plateau in the process of the origin and evolution of cultivated barley has attracted increasing attention^26^,^27^,^28^,^29^,^30^,^31^,^32^. Morphological, archaeological, cytogenetic and isozyme data revealed that wild barley on the Qinghai-Tibet Plateau is different from that in the Fertile Crescent^33^. Diversity array technology (DArT) data and population-based phylogenetic analyses indicated that the Tibetan Plateau and its vicinity is one of the domestication centers of cultivated barley^32^,^33^. Recent transcriptome profiling and population-based genetic diversity analysis also provided strong evidence that barley domestication may have occurred independently in geographically distinct regions^34^,^35^. However, in comparison to the abundant works on the Fertile Crescent and Central Asia, an Eastern center of origin and domestication of barley has long been underestimated^32^. Additional evidence is still needed to shed further light on cultivated barley domestication, in particular, the position of Qinghai-Tibet Plateau wild barley in origin and domestication events.

The varied evolutionary histories of wild barleys and widely dispersed landraces have generated diverse ecotypes, due to natural or human selection, resulting in a wide range of phenotypic/genotypic characteristics^36^,^37^,^38^. Over recent years molecular population genetics has been widely used to investigate genetic diversity within and among barley populations, and to trace the population structure and domestication events^22^,^36^,^39^,^40^,^41^,^42^. However, few investigations have been undertaken to examine genetic differentiation of barley on a worldwide scale, and, particularly, in relation to understanding geographic expansion and introgression.

Resequencing candidate genes can identify all mutations in a particular gene, thus allowing population-based analyses of genetic variation^43^. Recent advances in the phylogenetic and domestication history analysis with specific resequencing on multiple loci have been widely available in many crops^25^,^36^,^44^,^45^,^46^. However, not all genes reflect the history of a crop accurately. Although the majority of the genes in the genome will represent the true history of a domesticated lineage, domestication genes might falsely indicate incorrect origin^47^. Single copy nuclear genes hold a great potential to improve the robustness of phylogenetic reconstruction at all taxonomic levels, especially when universal markers such as cpDNA and/or nrDNA, are unable to generate strong phylogenetic hypotheses^48^. Single-copy nuclear genes are advantageous for studying the origin and phylogeny of species because of their high content of functional information and a modest rate of evolutionary change^48^,^49^. In this work, population-based resequencing and phylogenetic analysis of the second largest subunit of RNA polymerase II (*RPB2*) were performed. Nuclear RNA polymerases in eukaryotes have three distinct classes, which are frequently referred to as RNA polymerase I, II, and III. Each enzyme is composed of two large (>100 kDa) and several smaller subunits, each of which is typically encoded by a unique single-copy gene^50^. *RPB2* encodes the second-largest subunit of nuclear RNA polymerase II, which forms a part of the catalytic core that is believed to function in nucleotide binding and RNA chain elongation, and is responsible for the transcription of protein-encoding genes^51^,^52^. The only complete *RPB2* sequence in plants has been identified in *Arabidopsis thaliana*, which is 3,564 bp in length with 24 introns^53^. This gene is found in all eukaryotes, and large regions are highly conserved^50^. It has been demonstrated that *RPB2* is encoded by a single gene in many organisms, including *H. vulgare*^52^. A high level of polymorphisms present in this gene indicated that *RPB2* is an excellent tool in investigating molecular evolution and phylogenetic relationships^54^,^55^,^56^.

Understanding the origin of crops is important for exploiting elite genetic resources, and in helping to illuminate the history of domestication that would explain further the origin and development of modern cultivation and agronomy^2^. However, as mentioned above, the pattern of barley domestication is still controversial, information on geographically based genetic differentiation of barley populations on the worldwide scale is poorly documented, and how gene pool exchanges during spread and subsequent development of barley cultivation in the world remains to be explored. We used the *RPB2* gene to analyze the genetic variation among geographically distinct barley populations distributed worldwide. The objectives of our study were (i) to investigate genetic differentiation among wild barleys from the Near East Fertile Crescent and Tibetan Plateau populations, and between wild barley and cultivated barley sourced from different geographical regions; (ii) to address contentious points of barley domestication; and (iii) to examine introgression among worldwide barley populations.

## Results

### Haplotype analysis in barley populations

Of the 212 genotypes screened, 21 distinguishable haplotypes were identified. Haplotype compositions and frequencies in three wild barley populations and six cultivated barley populations were summarized in Table 1. A total of 21 haplotypes were identified in the 88 wild barley accessions, of which 18 haplotypes were identified in the Southwest Asian, 5 in the Central Asian and 4 in the Tibetan wild barley populations. Eighteen out of the 21 haplotypes were population specific: 15 specific to the Southwest Asian, 2 specific to the Central Asian and one specific to the Tibetan wild barley population. Only 6 haplotypes were identified in 124 domesticated lines, all 6 were present in the East Asian cultivated barley population, 5 and 4 in the Mediterranean and European cultivated barley population, respectively, and 3 in the remainder of the domesticated populations. However, no cultivated barley population specific haplotype was found. Haplotypes are shown in Supplementary Fig. S1. With the exception of the singleton polymorphisms (those occurring only once in the sample), 10 haplotype-specific SNPs were detected across 8 population-specific haplotypes. Of these, 8 SNPs were unique to the Southwest Asian wild population, and 2 each were unique to Central Asian and Tibetan wild barley.

**Figure 1:**
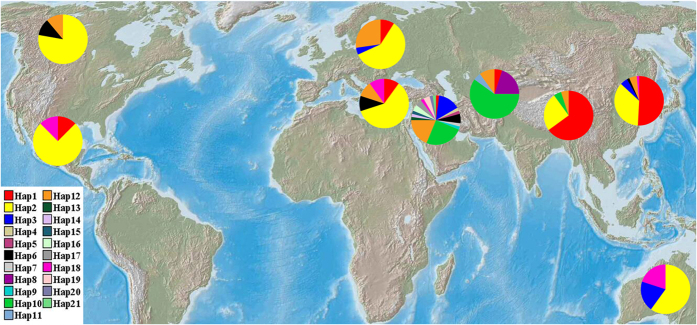
Geographic distribution of wild barley populations and cultivated barley populations, and *RPB2* haplotype frequencies among nine geographic regions. *RPB2* haplotype frequencies were displayed in pie diagrams and the proportion was given in percentage each with different color. The haplotype was calculated with DnaSP version 5.0^80^. The haplotype diagrams in Fig. 1 where generated by using SPSS 20.0 (IBM Corp., Armonk, NY, USA) and manually added to the map using Adobe Photoshop 9.0 (Adobe Systems Inc., San Jose, CA, USA). The original map was acquired from Google Maps (Map data: Google, NASA, TerraMetrics).

The haplotype frequencies present in all sampled accessions ranged from 0.005 to 0.325. Among all the haplotypes across the 212 accessions, 4 major haplotypes were detected. More than half of the accessions screened (119 of 212) have either haplotype Hap 1 or Hap 2, with Hap10 observed in 25 accessions (11.8%), and the Hap12 observed in 24 accessions (11.3%). The frequency of the other 17 haplotypes was low, ranging from 0.5% to 5.7%. *RPB2* haplotype frequencies differed markedly in different geographical populations. This was particularly evident for the haplotype Hap1, which was most frequent in Tibetan wild barleys and East Asian cultivars (0.65 and 0.508, respectively), but absent in North American and Australian cultivated barleys, and rarely present in the remaining five barley populations. Also noticeable was absence of the Hap10 in all cultivated populations, which was rare in the Tibetan wild barley population (0.05), but the most frequent in the Central Asian and Southwest Asian wild barley populations (0.60 and 0.25, respectively). These rare haplotypes were confined to specific geographical regions. i.e., of the 14 haplotypes that were present in <2% of the accessions sampled, 12 haplotypes were unique to the Southwest Asian wild barley population and 2 haplotypes to the Central Asian wild barley population (Table 1; Fig. 1).

### Genetic diversity analysis and neutrality test

As shown in Table 2, the highest number of haplotypes (H = 21) and highest number of segregating sites (S = 21), as well as the greatest per-site nucleotide diversity (θ = 0.00558 ± 0.00181), haplotype diversity (Hd = 0.747) and nucleotide diversity (π = 0.00307) were observed in wild barley, while 13.5% haplotype diversity (Hd) and 18.2% nucleotide diversity (π) reduction were found in cultivated barley. Both Tajima’s D, and Fu and Li’s statistics were positive for cultivated barley, but negative for wild barley. Fu and Li’s values were significant (P < 0.05) for wild barley. However, for cultivated barley, Tajima and Fu and Li’s neutrality tests did not significantly depart from neutrality.

To reveal domestication pressures acting on geographically distinct barley populations and the genetic differentiation among them, genetic analysis and the neutrality test in different populations were further performed (Table 3). The highest number of haplotypes (H = 18), highest haplotype diversity (Hd = 0.785), and greatest per-site nucleotide diversity (θ = 0.00575 ± 0.00203) were observed in the Southwest Asian wild barley population among the three wild barley populations. The nucleotide diversity as measured by π was 0.00342, ranging from 0.00098 in the Central Asian wild barley population to 0.00352 in the Mediterranean coast landrace. Both Tajima and Fu and Li’s neutrality tests were not significant (P > 0.05) in all six cultivated populations as well as in the Tibetan and Central Asian wild barley populations. Positive values for both tests were obtained from the North American and European cultivated populations, as well as from Tibetan wild barley population. In contrast, both negative values were obtained from the East Asian, South American, Mediterranean Coast and Australian cultivated populations. However, Southwest Asian wild barley population showed significant negative Fu and Li’s D and F values (P < 0.05) (−2.52062 and −2.68559, respectively).

### Sequence polymorphism analysis

**Figure 2:**
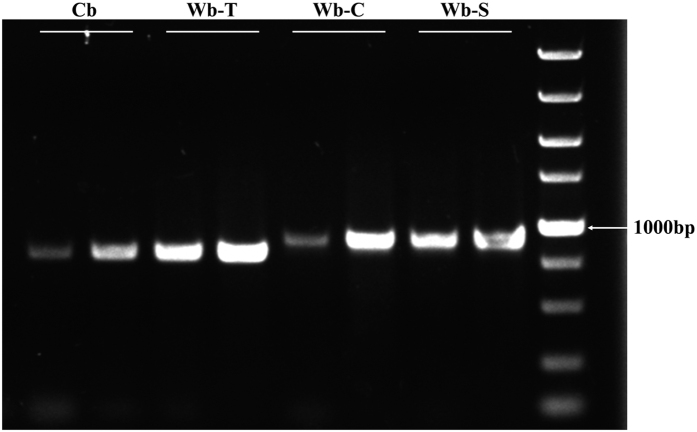
Example of an amplified pattern of the *RPB2* gene from three wild barley populations and sampled cultivated barleys. Wb-S (Wild barley of Southwest Asia), Wb-C (Wild barley of Central Asia), Wb-T (Wild barley of Tibet) and Cb (sampled cultivated barley).

The amplified *RPB2* fragments ranged from 745 bp to 858 bp in size. Its structure was further identified according to the published sequence of *H. vulgare* cDNA (GenBank accession number AF020839) in NCBI (http://www.ncbi.nlm.nih.gov/) (Supplementary Fig. S2). The example of amplified pattern of *RPB2* is shown in Fig. 2. Among three wild barley populations, amplicons with size of ~850 bp were detected in 95% of Central Asian wild barley accessions and 71% of Southwest Asian wild barley accessions, but in only 10% accessions of Tibetan wild barley.

**Figure 3:**
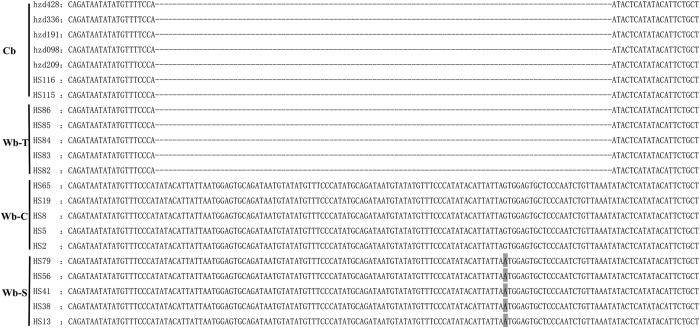
Partial alignment of the amplified sequences of *RPB2* from wild and cultivated barleys. Dashed lines indicate the deletion sequence. The bases with gray color indicate the single-base mutation compared to the same position of other samples. The accessions belongs to Wb-T (Wild barley of Tibet), Wb-C (Wild barley of Central Asia) and Wb-S (Wild barley of Southwest Asia) and Cb (sampled cultivated barley).

Multiple sequence alignments showed that a major of 105-bp deletion was clearly observed in the Tibetan wild barley and most cultivated accessions (108 of 124 accessions) (Fig. 3). However, the deletion in this region was rarely occurred in the Southwest Asian and Central Asian wild barley.

### Phylogenetic and STRUCTURE analysis

**Figure 4:**
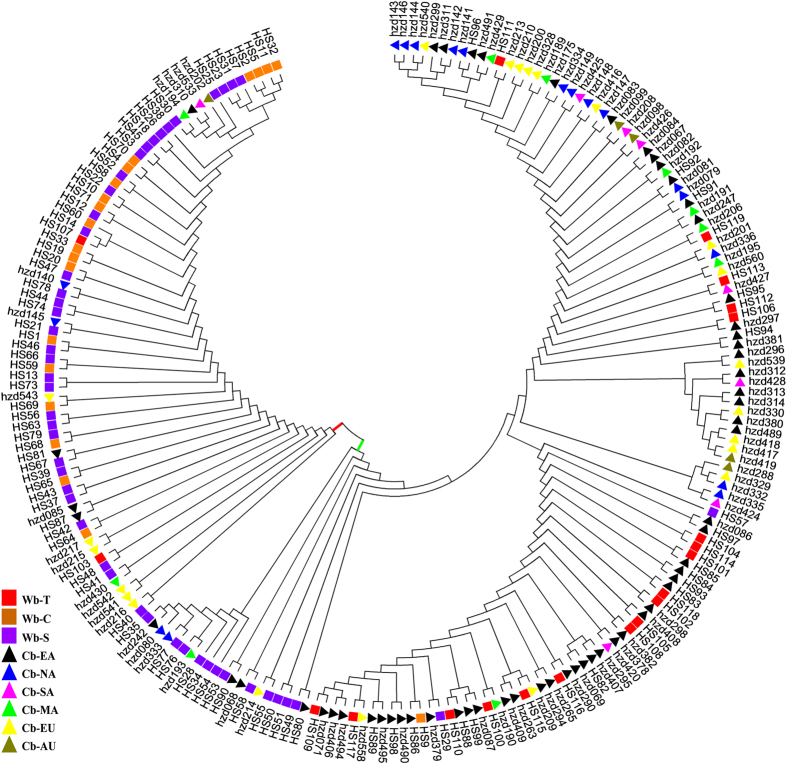
Phylogenetic tree (neighbor-joining) of 212 total barley accessions based on the *RPB2* gene. Two clusters, one comprised of a majority of wild barley accessions (represented in red bar) and another comprised of a majority of cultivated barley accessions (represented in green bar) are separated. The square stands for wild barley accessions: Tibet (Wb-T, red), Southwest Asia (Wb-S, purple), and Central Asia (Wb-C, orange), respectively; the triangle indicates cultivated barleys: East Asia (Cb-EA, black), North America (Cb-NA, blue), South America (Cb-SA, pink), Mediterranean Coast Areas (Cb-MA, green), Europe (Cb-EU, yellow), and Australia (Cb-AU, brown).

Multi-method phylogenetic analyses generated nearly identical topologies (data not shown). Neighbor-joining tree based on Tajima-Nei distance was shown here. Phylogenetic analysis of wild barley showed a separation of the Tibetan wild barleys (cluster I) from the most of Near East and Central Asian wild barleys (cluster II) (Supplementary Fig. S3). All 212 accessions were divided into two clusters (Fig. 4). The first contained the majority of wild barley accessions (red bar in Fig. 4) and the second cluster contained the majority of cultivated barley accessions (green bar in Fig. 4). However, the most of Tibetan wild barleys (18 of 20 accessions) and some Southwest Asian wild barleys (14 of 48 accessions) were distinct from the wild-dominated cluster, and appeared in the cultivars-dominated cluster.

**Figure 5:**
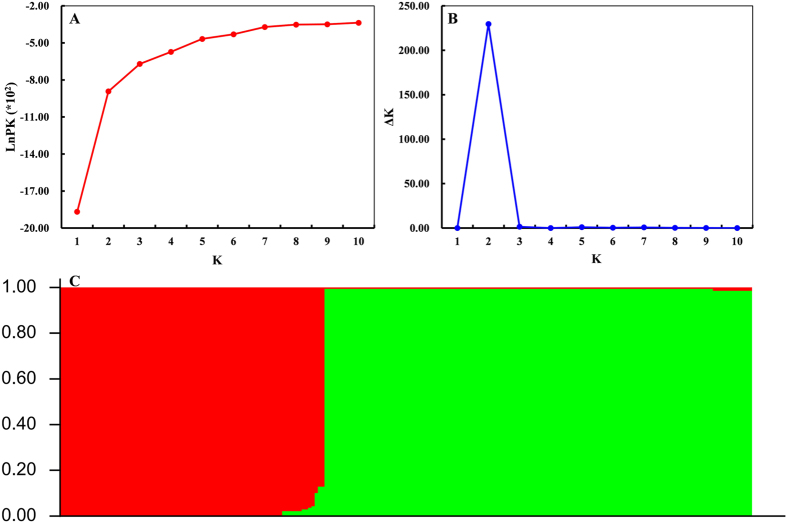
Analysis of population structure of 212 total barley accessions using STRUCTURE. (**A**) Estimated LnP(*K*) of possible clusters (*K*) from 1 to 10. (**B**) Δ*K* based on rate of change of LnP (*K*) between successive *K* values. (**C**) Population structure based on *K* = 2. Red represents Subgroup Q1; green, Subgroup Q2. Each part of figure was manually combined using Adobe Photoshop 9.0 (Adobe Systems Inc., San Jose, CA, USA).

STRUCTURE analysis revealed a clear evolutionary divergence between Near East and Tibetan wild barley (Supplementary Fig. S4). About 90% Tibetan wild barleys (18 of 20) with high membership coefficients of Q ≥ 0.991 were assigned to the population 1 (Q1 in Supplementary Fig. S4C), while 83.3% Southwest Asian wild barley (40 of 48) and 95% Central Asian wild barley accessions (19 of 20) were assigned to the population 2 (Q2 in Supplementary Fig. S4C), with membership coefficients of Q from 0.828 to 0.997. Structure analysis of all 212 barley accessions detected two groups, a wild-dominated group (Q1 in Fig. 5C) consisted mainly of most of wild barley accessions, and an admixed group (Q2 in Fig. 5C), which contained the most of cultivated barley, some Tibetan and Southwest Asian wild barleys. Cluster and structure analysis were also performed for 124 cultivated barley (data not shown): however, no visible subpopulation feather were recognized, which showed an admixed state, shown in Figs 4 and 5. All of the information of the STRUCTURE analysis results and inferred ancestry of individuals were shown in Supplementary Tables S1 and S2.

## Discussion

### Genetic differentiation among wild barley populations

Previous studies have provided evidence demonstrating a clear genetic differentiation among wild barley populations from Eastern and Central Asia with those from Near East areas^8^,^18^,^21^,^57^,^58^,^59^,^60^,^61^. Significant differentiation in roughly half of the sequenced loci from wild barley occurred between the Oriental and Occidental portion of the species^22^,^59^,^62^. Using resequencing data, Morrell and Clegg^22^ identified the differences in haplotype frequency at multiple loci between Fertile Crescent and Central Asian wild barley. Fang *et al*.^61^ recently found a strong genetic differentiation between the Eastern and Western populations on 2H and 5H. Previous morphological, distributional, archaeological, cytogenetic, and isozyme studies have also demonstrated that Tibetan wild barley was different from the Fertile Crescent samples^33^, which was also supported by the genome-wide DArT data^32^, transcriptome profiling^34^, and population-based genetic diversity analysis^35^. The current results showed significant genetic differentiation among wild barley populations. The distinct haplotype composition and obvious sequence variation were detected among Tibetan wild barley, Central Asian wild barley, and Southwest Asian wild barley (Table 1; Figs 1, 2 and 3; Supplementary Figs S1 and S2). Our phylogenetic analysis and population structure analysis also showed a certain degree of separation among Tibetan, Southwest Asian, and Central Asian wild barleys (Supplementary Figs S3 and S4). Our results provided further evidence to support multiple origination hypothesis of cultivated barley^21^,^22^,^32^, favoring that the wild barley domestication occurred in multiple geographically distinct regions.

### Tibet is a domestication center of cultivated barley

Since the discovery of *H. agriocrithon* E. Åberg, a close wild relative of barley, and of numerous *H. spontaneum* on the Qinghai-Tibet Plateau, the position of wild barley from the Tibetan Plateau in the process of origin and domestication of cultivated barley has received more attention and debate^33^. Extensive studies have reported that Tibetan wild barley was clearly different from other areas, and suggested that the Tibetan Plateau and its vicinity are the center of origin for cultivated barley in the Oriental region^29^,^30^,^31^,^40^, which was also supported by our data here. This was particularly evident for the haplotype Hap1, which was most frequent in the Tibetan wild barleys and East Asian cultivars (0.65 and 0.508, respectively), and haplotype Hap2 unique to Tibetan wild barley, which was also present in the most accessions of East Asian cultivated barleys (Table 1). Furthermore, multiple sequence alignments revealed a 105-bp deletion occurred in most accessions of Tibetan wild barleys, which also occurred in up to 95% of East Asian cultivars (Figs 2 and 3). Consequently, our results suggested that the East Asian cultivated barley might be evolved from the Tibetan wild barley, which is consistent with the report that barley landraces reflect a pattern of over shared ancestry with geographically proximate wild barley populations^63^. The present data thus provided further evidence to support the hypothesis that that Tibetan wild barley was the ancestor of Oriental domesticated barley^33^,^64^.

Our results not only merely confirmed that Tibetan wild barley contributed largely to East Asian cultivars as demonstrated above, but also revealed that these wild germplasms have important contribution to the cultivated barley gene pools outside the Oriental region. The haplotype analysis showed that the cultivars outside East Asia shared the same haplotypes with the wild barley from the Tibet (Table 1; Fig. 1). Sequence comparisons, phylogenetic and population structure analyses also revealed a close relationship between worldwide domesticated barley and the Tibetan wild barley (Figs 2, 3, 4 and 5). Our data confirmed that Tibetan Plateau is one of the centers of domestication of cultivated barley^32^,^34^,^35^.

### Multiple domestication and introgression of modern worldwide barleys

Hypotheses of the origin of barley have indicated that if the wild progenitor showed significant difference in allele frequencies among geographical regions, allelic composition is especially likely to be informative as to the number and locations of origin of domesticates^22^. For wild barley, the region with the highest level of genetic diversity is also most likely center of origin for the cultivated one^42^. In our study, highest number of haplotypes, greatest haplotype diversity and per-site nucleotide diversity were observed in the Southwest Asian wild barley population, which thus further confirmed that the Near East Fertile Crescent is a primary origin center of cultivated barley (Table 3). Additionally, the distinct haplotypes were detected not only in Southwest Asian wild barley, but also in Tibetan and Central Asian wild barleys (Table 1; Fig. 1). A great difference among distinct wild barleys, and a close relationship between these wild barleys and domesticated barley were revealed in our study, suggesting that Southwest Asian, Central Asian, and Tibetan wild barley are the ancestors of cultivars. Our results thus supported multiple origins of cultivated barley^22^,^32^.

In addition, the haplotypes analysis revealed that a significant proportion of the genetic composition of Eastern and Western wild barley has spread cultivars in other regions of the world. For example, haplotypes unique to Eastern wild barley (from Tibetan wild barley population) were also present in Occidental landraces, and haplotypes private to Western wild barley (from Southwest Asian wild barley population) were also found in Oriental landraces (Table 1; Fig. 1). As we observed, previous studies also reported that a significant proportion of Western genetic composition appeared in Indian and East Asian barleys, and the Eastern alleles were also found in Occidental landraces^25^,^32^,^65^. It was suggested that Central Asia is the sole route for wild barley migration between the Near East and the Tibetan Plateau^32^, as inferred in our haplotypes analysis; Hap1, Hap10 and Hap12 were shared among three wild barley populations and are most frequent in the Tibetan or Southwest Asian wild barleys, while rare in Central Asian wild barley population (Table 1; Fig. 1).

Consequently, our study provides new perspective on barley domestication and worldwide cultivation. We suggested that worldwide introgression has occurred following multiple domestication events, and, in this process Near East and Tibetan wild barleys have contributed to the modern cultivated barley gene pool.

Our scenario on barley origin and domestication may also offer an alternative explanation on why high genetic diversity and numbers of private haplotypes were present in Near East wild barley (Table 1; Table 3), and why specific haplotypes in Tibetan wild barley seem more widely present in cultivars at some locations and a close relationship between Tibetan wild and cultivated barley, as shown in previous reports^32^,^33^,^35^, as well in this study (Table 1; Fig. 1). Firstly, Near East *Hordeum spontaneum* is widely distributed as wild populations but largely isolated from cultivated barley^1^,^3^,^9^. However, wild barley in Tibet always coexists as a weed with cultivated barley and other field crops^27^, allowing gene flow to occur more easily between the two^32^. A long period of gene flow may have led to subsequent transfer of introgressed haplotypes to cultivars in other regions due to human activities such as germplasm exchange, introduction and hybridization^35^.

### Natural variation in the barley population

Domestication is the outcome of a selection process that led to increased adaptation to cultivation and utilization by humans^2^. Gene pools undergoing domestication experienced dramatic changes in allele frequencies due to genetic bottleneck and drift or selection, and some allelic combinations may be lost^37^,^38^,^66^. As expected, in this study, among the 21 haplotypes of *RPB2* sequence found in 212 barley accessions, only eight were present in the domesticated lines (Table 2), which agreed well with previous reports^33^,^35^,^67^, indicating that domesticated lines have lost most alleles in wild types^7^,^33^,^68^,^69^. About 18.2% nucleotide diversity, 13.5% haplotype diversity and two-fold of per-site nucleotide diversity reduction in cultivated barley, which is consistent with the studies such as Fu^43^ and Morrell *et al*.^25^, suggested that barley landraces might have suffered a population bottleneck during domestication and resulted in a reduction in genetic diversity^68^. Genetic bottleneck due to domestication and breeding is the major determinant of polymorphism loss in the domesticated lines sampled^67^. This loss is evident in a shift toward more positive values of Tajima’s D in the domesticated relative to wild populations^25^,^35^. Similarly, in our study, positive values of Tajima’s D and Fu, and Li’s were found in cultivated barley, while negative values were found in wild barley (Table 2). This is consistent with previous studies^70^,^71^ and supports that genetic bottleneck tends to result in a loss of rare variants^72^. *RPB2* showed significant negative values of Fu and Li’s D and F when all wild barley were considered (Table 2); this could potentially indicate a deviation from neutrality, possibly due to positive selection^67^. However, both Tajima’s D, and Fu and Li’s values in all cultivated barley were insignificant positive, which may have resulted from balancing selection or bottleneck effect.

It was notable that the genetic diversity in some domesticated barley populations was higher than that in wild barley populations, which is consistent with previous observations of the same gene in *Vitis vinifera*^56^, but in contrast with what we have demonstrated above that the gene pool of whole cultivated barley suffered a reduction in genetic diversity. We suggest that there are two possible explanations. Firstly, this might be caused by the nature of the *RPB2* gene, as it encodes the second largest subunit of nuclear RNA polymerase II, and is responsible for the transcription of protein encoding genes, which are very important for various aspects of plant life^54^. The different barley populations are from diverse environments, which could increase selection pressure on *RPB2*. The second explanation is the higher genetic variability and the higher substitution rate of *RPB2* in the domesticated barley as suggested by Zecca and Grassi^56^, can be viewed as a consequence of natural conditions, human selection, and germplasm exchange and breeding. Tajima’s D, and Fu and Li’s values in cultivated populations vary from positive to negative, indicating that distinct geographical and environmental barley population may be subjected to different selective pressure (Table 3). Balancing selection or bottleneck may act upon North American and European cultivated barley populations where rare-allele advantage resulted in an accumulating allelic frequency up to an intermediate level that may have caused a positive value of Tajima’s D, as suggested by Chung *et al*.^73^. However, purifying selection might act on the remaining domesticated barley populations, reflecting a negative statistical values in these regions^68^. In this study, Tajima’s D, and Fu and Li’s neutrality tests revealed no evidence of natural selection for Tibetan wild barley population, but under purifying selection as revealed by a high statistic positive value. This insignificant result may be attributed to the low polymorphism observed, which weakens the neutrality test. This result agrees with previous reports on *CPsHSP-2* in *Machilus kusano*^73^. Obviously, deviation from neutrality with Fu and Li’s values was significant (P < 0.05) for Southwest Asian wild barley population, which resulted from the observed number of rare variants that exceeded the expected number in an equilibrium neutral model and could be interpreted as being a result of a selective sweep or a population expansion^73^.

In summary, our study provided new insights into the origin and domestication of worldwide cultivated barley. The current results showed a clear genetic differentiation among Tibetan, Southwest Asian and Central Asian wild barleys. Tibetan Plateau is one of the domestication centers of cultivated barley. Our data suggested that multiple domestication followed by extensive introgression among modern worldwide cultivated barley. Moreover, our data showed divergent domestication pressures acting on geographically discontinuous barley populations.

## Methods

### Plant Materials

A total of 212 barley accessions were used in this study including 88 wild barley (*Hordeum spontaneum*) accessions from different geographic origins and 124 worldwide cultivated barley (*Hordeum vulgare*) accessions. The wild barley populations included: 48 wild barley accessions from the Southwest Asia (Israel, Jordan, Ethiopia, Lebanon, Azerbaijan, Syria, Iraq, and Turkey); 20 wild barley accessions from Central Asia (Iran, Afghanistan, Pakistan, and Tajikistan); and 20 wild barley from Qinghai-Tibet Plateau. One hundred and twenty-four accessions of cultivated barley were collected from 18 countries: 61 from Eastern Asia, 8 from South America, 18 form North America, 10 from Mediterranean coast areas, 5 form Australia, and 22 from Europe. Those materials were provided by the USDA (United States Department of Agriculture) and Huazhong Agricultural University barley germplasm collection. Information on accession numbers, and geographical origins of individuals used in this investigation are given in Supplementary Table S3.

### DNA extraction, *RPB2* gene amplification and sequencing

The seeds were planted in pots with nutrient soil, and maintained in a growth chamber with 14 h of light at 22 °C and 10 h of darkness at 18 °C prior to DNA extraction. Young leaves were collected from 5 to 10 plants of each accession. Total genomic DNA was isolated from freeze-dried leaf tissue following the cetyltrimethylammonium bromide (CTAB) extraction method of Stein *et al*.^74^. The quality of DNA was checked using 0.8% agarose gel electrophoresis, and further measured using spectrophotometer. The *RPB2* gene sequences were amplified using polymerase chain reaction (PCR) with primer P6F (5′-TGGGGAATGATGTGTCCTGC-3′) and P6FR (5′-CGAACCACACCAACTTCAGTGT-3′)^54^. PCR amplification was performed in Bio-Rad iCycler thermal cycler (Bio-Rad, USA). Each PCR reaction mixture (40 μl) consisting of 60 ng template DNA, 0.2 μM of each primer, 1.5 mM MgCl_2_, 0.2 mM of each deoxynucleotide (dATP, dCTP, dGTP, dTTP), 1.5 unit of high-fidelity polymerase ExTaq (TaKaRa, Dalian, China), and distilled deionized water was added to make up the final volume of 40 μl. The PCR was programmed at an initial denaturing of 4 min at 95 °C, followed by 40 cycles of 1 min at 95 °C, 1 min annealing temperatures at 56 °C, 2 min extension at 72 °C and a final extension step at 72 °C for 8 min.

The amplified products were separated by electrophoresis in 1% agarose gels, and the single specific PCR product band was purified by the QIAquick PCR purification kit (Qiagen, Germany) according to the manufacturer′s instruction. DNA was sequenced commercially at the Beijing Tsing Ke BioTech Co., Ltd (Beijing, China). To exclude sequencing errors induced by *Taq* DNA polymerase during PCR amplification, for each accession, the amplifying and sequencing were repeated three times. The final nucleotide sequence was determined from the sequencing results of both forward and reverse strands, and further data quality were checked using Chromas 2.32 (Technelysium Pty. Ltd.).

### Data Analysis

Multiple sequence alignments were performed using ClustalX^75^. Nucleotide diversity was estimated by Tajima′s π^76^ and Watterson’s^77^ statistics. Tests of neutral evolution were performed as described by Tajima^78^, and Fu and Li^79^. The above calculations were conducted using the software program DnaSP version 5.0^80^. Each insertion/deletion (indel) was considered as a single mutation event, and all indels were therefore coded as single positions. Identical sequences were grouped into haplotypes (Hap). Phylogenetic analysis was performed with the computer program MEGA 6^81^ using the maximum likelihood (ML) method under the Kimura 2-parameter model, the minimum-evolution (ME) and neighbor-joining (NJ) methods with the model of Tajima-Nei. The confidence of each clade was calculated based on the bootstrap values with 1,000 replications.

The population structure was analyzed using STRUCTURE software (version 2.3.4)^82^,^83^. Haplotypes were recoded as unique alleles. Multistep approach (after several trial runs) was applied to infer the genetic structure in our wild, cultivated as well as all barley samples, respectively. The first step of the analysis consisted of estimating *K*-value (the putative number of genetic groups). Twenty independent runs of *K* from 1 to 10 were performed, with 100,000 MCMC (Markov Chain Monte Carlo) iterations and a burn-in period of 50,000 replicates under the ‘admixture model’. The most likely *K*-value was estimated by the log probability of data [LnP(D)] and an ad hoc statistic Δ*K* based on the rate of change of LnP(D) between successive *K* values as described by Evanno *et al*.^84^. To infer the appropriate number of *K*, STRUCTURE HARVESTER^85^ (http://taylor0.biology.ucla.edu/structureHarvester/index.php) was used. In a second step, after the inference of *K*, the STRUCTURE procedure was repeated with a fixed *K* and 10 independent runs with 50,000 MCMC iterations and a burn-in period of 25,000. An individual was assigned to a certain cluster if its q value was higher than 0.75.

## Additional Information

**How to cite this article**: Wang, Y. *et al*. Molecular evidence of RNA polymerase II gene reveals the origin of worldwide cultivated barley. *Sci. Rep.* **6**, 36122; doi: 10.1038/srep36122 (2016).

**Publisher’s note:** Springer Nature remains neutral with regard to jurisdictional claims in published maps and institutional affiliations.

## Supplementary Material

Supplementary Information

## Figures and Tables

**Table 1:** Haplotype frequencies of *RPB2* gene in three wild barley populations and six cultivated barley populations.

RPB2	Wb-T (20)	Wb-C (20)	Wb-S (48)	Cb-EA (61)	Cb-NA (18)	Cb-SA (8)	Cb-MA (10)	Cb-EU (22)	Cb-AU (5)	Overall (212)
Hap1	0.65 (13)	0.05 (1)	0.021 (1)	0.508 (31)	0	0.125 (1)	0.10 (1)	0.091 (2)	0	0.236 (50)
Hap2	0.25 (5)	0	0	0.361 (22)	0.778 (14)	0.750 (6)	0.60 (6)	0.591 (13)	0.60 (3)	0.325 (69)
Hap3	0	0	0.146 (7)	0.049 (3)	0	0	0	0.045 (1)	0.20 (1)	0.057 (12)
Hap4	0	0	0.021 (1)	0	0	0	0	0	0	0.005 (1)
Hap5	0	0	0.021 (1)	0	0	0	0	0	0	0.005 (1)
Hap6	0	0	0.063 (3)	0.016 (1)	0.111 (2)	0	0.10 (1)	0	0	0.033 (7)
Hap7	0	0	0.021 (1)	0	0	0	0	0	0	0.005 (1)
Hap8	0	0.20 (4)	0	0	0	0	0	0	0	0.019 (4)
Hap9	0	0	0.021 (1)	0	0	0	0	0	0	0.005 (1)
Hap10	0.05 (1)	0.60 (12)	0.250 (12)	0	0	0	0	0	0	0.118 (25)
Hap11	0	0.05 (1)	0	0	0	0	0	0	0	0.005 (1)
Hap12	0.05 (1)	0.10 (2)	0.188 (9)	0.049 (3)	0.111 (2)	0	0.10 (1)	0.273 (6)	0	0.113 (24)
Hap13	0	0	0.021 (1)	0	0	0	0	0	0	0.005 (1)
Hap14	0	0	0.021 (1)	0	0	0	0	0	0	0.005 (1)
Hap15	0	0	0.021 (1)	0	0	0	0	0	0	0.005 (1)
Hap16	0	0	0.042 (2)	0	0	0	0	0	0	0.009 (2)
Hap17	0	0	0.042 (2)	0	0	0	0	0	0	0.009 (2)
Hap18	0	0	0.021 (1)	0.016 (1)	0	0.125 (1)	0.10 (1)	0	0.20 (1)	0.024 (5)
Hap19	0	0	0.042 (2)	0	0	0	0	0	0	0.009 (2)
Hap20	0	0	0.021 (1)	0	0	0	0	0	0	0.005 (1)
Hap21	0	0	0.021 (1)	0	0	0	0	0	0	0.005 (1)

The three wild populations are Wb-T (Wild barley of Tibet), Wb-C (Wild barley of Central Asia) and Wb-S (Wild barley of Southwest Asia) respectively; The six cultivated populations as follows: Cb-EA (Cultivated barley of East Asia), Cb-NA (Cultivated barley of North America), Cb-SA (Cultivated barley of South America), Cb-MA (Cultivated barley of the Mediterranean Coast Areas), Cb-EU (Cultivated barley of Europe) and Cb-AU (Cultivated barley of Australia).

**Table 2:** Estimates of genetic diversity and test statistics for selection at *RPB2* gene in wild and cultivated barley accessions.

Population	No. of accessions	No. of haplotypes (H)	No. of segregating sites (S)	Haplotype diversity (Hd)	Theta (per site) from S (θ)	Nucleotide diversity (π)	Tajima’s D test	Fu and Li’s D test	Fu and Li’s F test
**Wild barley**	88	21	21	0.747	0.00558 ± 0.00181	0.00307	−1.33098	−2.41720*	−2.40320*
**Cultivated barley**	124	6	8	0.646	0.00199 ± 0.00082	0.00251	0.61741	1.24423	1.21958
**All**	212	21	21	0.774	0.00475 ± 0.00143	0.00342	−0.75435	−2.21984	−1.96810

Note: the gaps/missing/data were excluded; *significant at 0.05 level.

**Table 3:** Estimates of nucleotide diversity per base pair and test statistics for selection at *RPB2* gene in different barley populations.

Population	No. of accessions	No. of haplotypes (H)	Haplotype diversity (Hd)	Theta (per site) from S (θ)	Nucleotide diversity (π)	Tajima’s D test	Fu and Li’s D test	Fu and Li’s F test
**Wb-T**	20	4	0.532	0.00187 ± 0.00101	0.00192	0.07112	1.18636	1.00914
**Wb-C**	20	5	0.416	0.00187 ± 0.00101	0.00098	−1.46008	−2.01240	−2.14351
**Wb-S**	48	18	0.731	0.00575 ± 0.00203	0.00254	−1.77877	−2.52062*	−2.68559*
**Cb-EA**	61	6	0.616	0.00229 ± 0.00099	0.00202	−0.31309	−0.93653	−0.86200
**Cb-NA**	18	3	0.392	0.00193 ± 0.00105	0.00202	0.13644	1.19899	1.04346
**Cb-SA**	8	3	0.464	0.00361 ± 0.00198	0.00258	−1.35929	−1.36041	−1.50298
**Cb-MA**	10	5	0.667	0.00379 ± 0.00196	0.00352	−0.31377	−0.38531	−0.41309
**Cb-EU**	22	4	0.593	0.00182 ± 0.00097	0.00256	1.20224	1.17564	1.36798
**Cb-AU**	5	3	0.700	0.00386 ± 0.00235	0.00348	−0.66823	−0.66823	−0.69243
**All**	212	21	0.774	0.00475 ± 0.00143	0.00342	−0.75435	−2.21984	−1.96810

The three wild populations are Wb-T (Wild barley of Tibet), Wb-C (Wild barley of Central Asia) and Wb-S (Wild barley of Southwest Asia) respectively; The six cultivated populations as follows: Cb-EA (Cultivated barley of East Asia), Cb-NA (Cultivated barley of North America), Cb-SA (Cultivated barley of South America), Cb-MA (Cultivated barley of the Mediterranean Coast Areas), Cb-EU (Cultivated barley of Europe) and Cb-AU (Cultivated barley of Australia). Note: the gaps/missing/data were excluded; *significant at 0.05 level.
