# Supplementary material for: Molecular evidence of RNA polymerase II gene reveals the origin of worldwide cultivated barley
Source: Sci Rep. 2016 Oct 27;6:36122. doi: 10.1038/srep36122 (PMC5081693; doi:10.1038/srep36122)
Supplement: Supplementary Information [file srep36122-s1.pdf]

Supplementary Information

Molecular evidence of RNA polymerase II gene reveals the origin of worldwide cultivated barley

Yonggang Wang, Xifeng Ren, Dongfa Sun*, Genlou Sun*

*** Correspondence:**
Dongfa Sun: sundongfa1@mail.hzau.edu.cn
Genlou Sun: genlou.sun@smu.ca

Supplementary Figure S1. A haplotype of *RPB2* gene in all barley accessions. The symbols within the sequence indicate indel polymorphisms (-), and weakly conserved (.). Singleton polymorphisms (those occurring only once in the sample) are excluded. The SNPs (indel included) that were unique to the wild barley of Tibet, Central Asia and Southwest Asia are highlighted in red, green and brown, respectively.

```
                   *         *         *         *         *         *         *         *         *         *         *         *         *
Hap_1     TTAAGTTCGCCACC---------------------------------------------------------------------------------------------------------ATGGTTAGGGGACAG
Hap_2     .............T---------------------------------------------------------------------------------------------------------............T..
Hap_3     ..............---------------------------------------------------------------------------------------------------------............T..
Hap_4     .....-...T....---------------------------------------------------------------------------------------------------------C...........T..
Hap_5     .........T....---------------------------------------------------------------------------------------------------------C...........T..
Hap_6     ...-.....T....---------------------------------------------------------------------------------------------------------CC..........T..
Hap_7     .........T....---------------------------------------------------------------------------------------------------------C.A.........T..
Hap_8     .......T.T....TATACATTATTAATGGAGTGCAGATAATGTATATGTTTCCCATATGCAGATAATGTATATGTTTCCCATATACATTATTAGTGGAGTGCTCCCAATCTGTTAAATC...........T.C
Hap_9     .........T....TATACATTATTAATGGAGTGCAGATAATGTATATGTTTCCCATATGCAGATAATGTATATGTTTCCCATATACATTATCAGTGGAGTGCTCCCAATCTGTTAAATC...........T.C
```

```
Hap_10    .........T....TATACATTATTAATGGAGTGCAGATAATGTATATGTTTCCCATATGCAGATAATGTATATGTTTCCCATATACATTATTAGTGGAGTGCTCCCAATCTGTTAAATC...........T.C
Hap_11    .........T....TATACATTATTAATGGAGTGCAGATACTGTATATGTTTCCCATATGCAGATAATGTATATGTTTCCCATATACATTATTAATGGAGTGCTCCCAATCTGTTAAATC...........T.C
Hap_12    .........T....TATACATTATTAATGGAGTGCAGATAATGTATATGTTTCCCATATGCAGATAATGTATATGTTTCCCATATACATTATTAATGGAGTGCTCCCAATCTGTTAAATC...........T.C
Hap_13    .C.......T....TATACATTATTAATGGAGTGCAGATAATGTATATGTTTCCCATATGCAGATAATGTATATGTTTCCCATATACATTATTAATGGAGTGCTCCCAATCTGTTAAATC...........T.C
Hap_14    ..G......T....TATACATTATTAATGGAGTGCAGATAATGTATATGTTTCCCATATGCAGATAATGTATATGTTTCCCATATACATTATTAATGGAGTGCTCCCAATCTGTTAAATC...........T.C
Hap_15    .........T....TATACATTATTAATGGAGTGCAGATAATGTATATGTTTCCCATATGCAGATAATGTATATGTTTCCCATATACATTATTAATGGAGTGCTCCCAATCTGTTAAATC...A.......T.C
Hap_16    .........T....TATACATTATTAATGGAGTGCAGATAATGTATATGTTTCCCATATGCAGATAATGTATATGTTTCCCATATACATTATTAATGGAGTGCTCCCAATCTGTTAAATC...........TGC
Hap_17    ......G..T.GA.TATACATTATTAATGGAGTGCAGATAATGTATATGTTTCCCATATGCAGATAATGTATATGTTTCCCATATACATTATTAATGGAGTGCTCCCAATCTGTTAAATC...........T.C
Hap_18    .........TA...TATACATTATTAATGGAGTGCAGATAATGTATATGTTTCCCATATGCAGATAATGTATATGTTTCCCATATACATTATTAATGGAGTGCTCCCAATCTGTTAAATC..A..------T.C
Hap_19    .........T....TATACATTATTAATGGAGTGCAGATAATGTATATGTTTCCCATATGCAGATAATGTATATGTTTCCCATATACATTATTAATGGAGTGCTCCCAATCTGTTAAATC.....------T.C
Hap_20    ....T....T....TATACATTATTAATGGAGTGCAGATAATGTATATGTTTCCCATATGCAGATAATGTATATGTTTCCCATATACATTATTAATGAAGTGCTCCCAATCTGTTAAATC.....------T.C
Hap_21    C.......AT....TATACATTATTAATGGAGTGCAGATAATGTATATGTTTCCCATATGCAGATAATGTATATGTTTCCCATATACATTATTAATGGAGTGCTCCCAATCTGTTAAATC....C......T.C
```

Supplementary Figure S2. Multiple sequence alignment of *RPB2* gene for different haplotypes. The sequences were compared to the reference sequence (GenBank accession number AF020839). The symbols under the sequence alignment indicate identical residues (*); A horizontal dash indicates the indel polymorphisms. Coding regions are shaded in gray.

Hap_1	GACATCAATCTGAAAATTAACAACATTCAGCATGTAATTTTGAGACCTGAATGTCGTTAA
Hap_2	GACATCAATCTGAAAATTAACAACATTCAGCATGTAATTTTGAGACCTGAATGTCGTTAA
Hap_3	GACATCAATCTGAAAATTAACAACATTCAGCATGTAATTTTGAGACCTGAATGTCGTTAA
Hap_4	GACATCAATCTGAAAATTAACAACATTCAGCATGTAATTTTGAGACCTGAATGTCGTTAA
Hap_5	GACATCAATCTGAAAATTAACAACATTCAGCATGTAATTTTGAGACCTGAATGTCGTTAA
Hap_6	GACATCAATCTGAAAATTAACAACATTCAGCATGTAATTTTGAGACCTGAATGTCGTTAA
Hap_7	GACATCAATCTGAAAATTAACAACATTCAGCATGTAATTTTGAGACCTGAATGTCGTTAA
Hap_8	GACATCAATCTGAAAATTAACAACATTCAGCATGTAATTTTGAGACCTGAATGTCGTTAA
Hap_9	GACATCAATCTGAAAATTAACAACATTCAGCATGTAATTTTGAGACCTGAATGTCGTTAA
Hap_10	GACATCAATCTGAAAATTAACAACATTCAGCATGTAATTTTGAGACCTGAATGTCGTTAA
Hap_11	GACATCAATCTGAAAATTAACAACATTCAGCATGTAATTTTGAGACCTGAATGTCGTTAA
Hap_12	GACATCAATCTGAAAATTAACAACATTCAGCATGTAATTTTGAGACCTGAATGTCGTTAA
Hap_13	GACATCAATCTGAAAATTAACAACATTCAGCATGTAATTTTGAGACCTGAATGTCGTTAA
Hap_14	GACATCAATCTGAAAATTAACAACATTCAGCATGTAATTTTGAGACCTGAATGTCGTTAA
Hap_15	GACATCAATCTGAAAATTAACAACATTCAGCATGTAATTTTGAGACCTGAATGTCGTTAA
Hap_16	GACATCAATCTGAAAATTAACAACATTCAGCATGTAATTTTGAGACCTGAATGTCGTTAA
Hap_17	GACATCAATCTGAAAATTAACAACATTCAGCATGTAATTTTGAGACCTGAATGTCGTTAA
Hap_18	GACATCAATCTGAAAATTAACAACATTCAGCATGTAATTTTGAGACCTGAATGTCGTTAA
Hap_19	GACATCAATCTGAAAATTAACAACATTCAGCATGTAATTTTGAGACCTGAATGTCGTTAA
Hap_20	GACATCAATCTGAAAATTAACAACATTCAGCATGTAATTTTGAGACCTGAATGTCGTTAA
Hap_21	GACATCAATCTGAAAATTAACAACATTCAGCATGTAATCTTGAGACCTGAATGTCGTTAA
	************************************** *********************

Hap_1	AATCACATGCCGGCAATAAATGACTTTCTTAACATGTTCACCATACATCTTGAAAATGAT
Hap_2	AATCACATGCCGGCAATAAATGACTTTCTTAACATGTTCACCATACATCTTGAAAATGAT
Hap_3	AATCACATGCCGGCAATAAATGACTTTCTTAACATGTTCACCATACATCTTGAAAATGAT
Hap_4	AATCACATGCCGGCAATAAATGACTTTCTTAACATGTTCACCATACATCTTGAAAATGAT
Hap_5	AATCACATGCCGGCAATAAATGACTTTCTTAACATGTTCACCATACATCTTGAAAATGAT
Hap_6	AATCACATGCCGGCAATAAATGACTTTCTTAACATGTTCACCATAC-TCTTGAAAATGAT
Hap_7	AATCACATGCCGGCAATAAATGACTTTCTTAACATGTTCACCATACATCTTGAAAATGAT
Hap_8	AATCACATGCCGGCAATAAATGACTTTCTTAACATGTTCACCATACATCTTGAAAATGAT
Hap_9	AATCACATGCCGGCAATAAATGACTTTCTTAACATGTTCACCATACATCTTGAAAATGAT
Hap_10	AATCACATGCCGGCAATAAATGACTTTCTTAACATGTTCACCATACATCTTGAAAATGAT
Hap_11	AATCACATGCCGGCAATAAATGACTTTCTTAACATGTTCACCATACATCTTGAAAATGAT
Hap_12	AATCACATGCCGGCAATAAATGACTTTCTTAACATGTTCACCATACATCTTGAAAATGAT
Hap_13	AATCACATGCCGGCAATAAATGACCTTCTTAACATGTTCACCATACATCTTGAAAATGAT
Hap_14	AATCACATGCCGGCAATAAATGACTTTCTTAACATGTTCACCGTACATCTTGAAAATGAT
Hap_15	AATCACATGCCGGCAATAAATGACTTTCTTAACATGTTCACCATACATCTTGAAAATGAT
Hap_16	AATCACATGCCGGCAATAAATGACTTTCTTAACATGTTCACCATACATCTTGAAAATGAT
Hap_17	AATCACATGCCGGCAATAAATGACTTTCTTAACATGTTCACCATACATCTTGAAAATGAT
Hap_18	AATCACATGCCGGCAATAAATGACTTTCTTAACATGTTCACCATACATCTTGAAAATGAT
Hap_19	AATCACATGCCGGCAATAAATGACTTTCTTAACATGTTCACCATACATCTTGAAAATGAT
Hap_20	AATCACATGCCGGCAATAAATGACTTTCTTAACATGTTCACCATACATCTTTAAAATGAT
Hap_21	AATCACATGCCGGCAATAAATGACTTTCTTAACATGTTCACCATACATCTTGAAAATGAT
	************************ ***************** *** **** ********

```
Hap_1               CTTTACTTTGCATAGTAAGTACCAGATATCAAACTAATTCAGGATTGCATATGAAAGGGC
Hap_2               CTTTACTTTGCATAGTAAGTACCAGATATCAAACTAATTCAGGATTGCATATGAAAGGGC
Hap_3               CTTTACTTTGCATAGTAAGTACCAGATATCAAACTAATTCAGGATTGCATATGAAAGGGC
Hap_4               CTT-ACTTTGCATAGTAAGTACCAGATATCAAACTAATTCAGGATTGCATATGAAAGGGC
Hap_5               CTTTACTTTGCATAGTAAGTACCAGATATCAAACTAATTCAGGATTGCATATGAAAGGGC
Hap_6               CTTTACTTTGCATAGTAAGTACCAGATATCAAACTAATTCAGGATTGCATATGAAAGGGC
Hap_7               CTTTACTTTGCATAGTAAGTACCAGATATCAAACTAATTCAGGATTGCATATGAAAGGGC
Hap_8               CTTTACTTTGCATAGTAAGTACCAGATATCAAATTAATTCAGGATTGCATATGAAAGGGC
Hap_9               CTTTACTTTGCATAGTAAGTACCAGATATCAAACTAATTCAGGATTGCATATGAAAGGGC
Hap_10              CTTTACTTTGCATAGTAAGTACCAGATATCAAACTAATTCAGGATTGCATATGAAAGGGC
Hap_11              CTTTACTTTGCATAGTAAGTACCAGATATCAAACTAATTCAGGATTGCATATGAAAGGGC
Hap_12              CTTTACTTTGCATAGTAAGTACCAGATATCAAACTAATTCAGGATTGCATATGAAAGGGC
Hap_13              CTTTACTTTGCATAGTAAGTACCAGATATCAAACTAATTCAGGATTGCATATGAAAGGGC
Hap_14              CTTTACTTTGCATAGTAAGTACCAGATATCAAACTAATTCAGGATTGCATATGAAAGGGC
Hap_15              CTTTACTTTGCATAGTAAGTACCAGATATCAAACTAATTCAGGATTGCATATGAAAGGGC
Hap_16              CTTTACTTTGCATAGTAAGTACCAGATATCAAACTAATTCAGGATTGCATATGAAAGGGC
Hap_17              CTTTACTTTGCAGAGTAAGTACCAGATATCAAACTAATTCAGGATTGCATATGAAAGGGC
Hap_18              CTTTACTTTGCATAGTAAGTACCAGATATCAAACTAATTCAGGATTGCATATGAAAGGGC
Hap_19              CTTTACTTTGCATAGTAAGTACCAGATATCAAACTAATTCAGGATTGCATATGAAAGGGC
Hap_20              CTTTACTTTGCATAGTAAGTACCAGATATCAAACTAATTCAGGATTGCATATGAAAGGGC
Hap_21              CTTTACTTTGCATAGTAAGTACCAGATATCAAACTAATTCAGGATTGCATATAAAAGGGC
                    *** ******** ******************** ****************** *******

Hap_1               AACAAACCACATCAGTTTTATTAATGGAGTGCTCCCAATCTGTTAAATATACAAAATATG
Hap_2               AACAAACCACATCAGTTTTATTAATGGAGTGCTCCCAATCTGTTAAATATACAAAATATG
Hap_3               AACAAACCACATCAGTTTTATTAATGGAGTGCTCCCAATCTGTTAAATATACAAAATATG
Hap_4               AACAAATCACATCAGTTTTATTAATGGAGTGCTCCCAATCTGTTAAATATACAAAATATG
Hap_5               AACAAATCACATCAGTTTTATTAATGGAGTGCTCCCAATCTGTTAAATATACAAAATATG
Hap_6               AACAAATCACATCAGTTTTATTAATGGAGTGCTCCCAATCTGTTAAATATACAAAATATG
Hap_7               AACAAATCACATCAGTTTTATTAATGGAGTGCTCCCAATCTGTTAAATATACAAAATATG
Hap_8               AACAAATCACATCAGTTTTATTAATGGAGTGCTCCCAATCTGTTAAATATACAAAATATG
Hap_9               AACAAATCACATCAGTTTTATTAATGGAGTGCTCCCAATCTGTTAAATATACAAAATATG
Hap_10              AACAAATCACATCAGTTTTATTAATGGAGTGCTCCCAATCTGTTAAATATACAAAATATG
Hap_11              AACAAATCACATCAGTTTTATTAATGGAGTGCTCCCAATCTGTTAAATATACAAAATATG
Hap_12              AACAAATCACATCAGTTTTATTAATGGAGTGCTCCCAATCTGTTAAATATACAAAATATG
Hap_13              AACAAATCACATCAGTTTTATTAATGGAGTGCTCCCAATCTGTTAAATATACAAAATATG
Hap_14              AACAAATCACATCAGTTTTATTAATGGAGTGCTCCCAATCTGTTAAATATACAAAATATG
Hap_15              AACAAATCACATCAGTTTTATTAATGGAGTGCTCCCAATCTGTTAAATATACAAAATATG
Hap_16              AACAAATCACATCAGTTTTATTAATGGAGTGCTCCCAATCTGTTAAATATACAAAATATG
Hap_17              AACAAATCACATCAGTTTTATTAATGGAGTGCTCCCGATCTGTTAAATATAAAAAATATG
Hap_18              AACAAATCACATCAGTTTTATTAATGGAGTGCTCACAATCTGTTAAATATACAAAATATG
Hap_19              AACAAATCACATCAGTTTTATTAATGGAGTGCTCCCAATCTGTTAAATATACAAAATATG
Hap_20              AACAAATCACATCAGTTTTATTAATGGAGTGCTCCCAATCTGTTAAATATACAAAATATG
Hap_21              AACAAATCACATCAGTTTTATTAATGGAGTGCTCCCAATCTGTTAAATATACAAAATATG
                    ****** *************************** * ************** ********

Hap_1               CAGATAATATATGTTTCCCA----------------------------------------
Hap_2               CAGATAATATATGTTTCCCA----------------------------------------
Hap_3               CAGATAATATATGTTTCCCA----------------------------------------
Hap_4               CAGATAATATATGTTTCCCA----------------------------------------
```

```
Hap_5               CAGATAATATATGTTTCCCA----------------------------------------
Hap_6               CAGATAATATATGTTTCCCA----------------------------------------
Hap_7               CAGATAATATATGTTTCCCA----------------------------------------
Hap_8               CAGATAATATATGTTTCCCATATACATTATTAATGGAGTGCAGATAATGTATATGTTTCC
Hap_9               CAGATAATATATGTTTCCCATATACATTATTAATGGAGTGCAGATAATGTATATGTTTCC
Hap_10              CAGATAATATATGTTTCCCATATACATTATTAATGGAGTGCAGATAATGTATATGTTTCC
Hap_11              CAGATAATATATGTTTCCCATATACATTATTAATGGAGTGCAGATACTGTATATGTTTCC
Hap_12              CAGATAATATATGTTTCCCATATACATTATTAATGGAGTGCAGATAATGTATATGTTTCC
Hap_13              CAGATAATATATGTTTCCCATATACATTATTAATGGAGTGCAGATAATGTATATGTTTCC
Hap_14              CAGATAATATATGTTTCCCATATACATTATTAATGGAGTGCAGATAATGTATATGTTTCC
Hap_15              CAGATAATATATGTTTCCCATATACATTATTAATGGAGTGCAGATAATGTATATGTTTCC
Hap_16              CAGATAATATATGTTTCCCATATACATTATTAATGGAGTGCAGATAATGTATATGTTTCC
Hap_17              CAGATAATATATGTTTCCCATATACATTATTAATGGAGTGCAGATAATGTATATGTTTCC
Hap_18              CAGATAATATATGTTTCCCATATACATTATTAATGGAGTGCAGATAATGTATATGTTTCC
Hap_19              CAGATAATATATGTTTCCCATATACATTATTAATGGAGTGCAGATAATGTATATGTTTCC
Hap_20              CAGATAATATATGTTTCCCATATACATTATTAATGGAGTGCAGATAATGTATATGTTTCC
Hap_21              CAGATAATATATGTTTCCCATATACATTATTAATGGAGTGCAGATAATGTATATGTTTCC
                    **************** ***

Hap_1               ------------------------------------------------------------
Hap_2               ------------------------------------------------------------
Hap_3               ------------------------------------------------------------
Hap_4               ------------------------------------------------------------
Hap_5               ------------------------------------------------------------
Hap_6               ------------------------------------------------------------
Hap_7               ------------------------------------------------------------
Hap_8               CATATGCAGATAATGTATATGTTTCCCATATACATTATTAGTGGAGTGCTCCCAATCTGT
Hap_9               CATATGCAGATAATGTATATGTTTCCCATATACATTATCAGTGGAGTGCTCCCAATCTGT
Hap_10              CATATGCAGATAATGTATATGTTTCCCATATACATTATTAGTGGAGTGCTCCCAATCTGT
Hap_11              CATATGCAGATAATGTATATGTTTCCCATATACATTATTAATGGAGTGCTCCCAATCTGT
Hap_12              CATATGCAGATAATGTATATGTTTCCCATATACATTATTAATGGAGTGCTCCCAATCTGT
Hap_13              CATATGCAGATAATGTATATGTTTCCCATATACATTATTAATGGAGTGCTCCCAATCTGT
Hap_14              CATATGCAGATAATGTATATGTTTCCCATATACATTATTAATGGAGTGCTCCCAATCTGT
Hap_15              CATATGCAGATAATGTATATGTTTCCCATATACATTATTAATGGAGTGCTCCCAATCTGT
Hap_16              CATATGCAGATAATGTATATGTTTCCCATATACATTATTAATGGAGTGCTCCCAATCTGT
Hap_17              CATATGCAGATAATGTATATGTTTCCCATATACATTATTAATGGAGTGCTCCCAATCTGT
Hap_18              CATATGCAGATAATGTATATGTTTCCCATATACATTATTAATGGAGTGCTCCCAATCTGT
Hap_19              CATATGCAGATAATGTATATGTTTCCCATATACATTATTAATGGAGTGCTCCCAATCTGT
Hap_20              CATATGCAGATAATGTATATGTTTCCCATATACATTATTAATGAAGTGCTCCCAATCTGT
Hap_21              CATATGCAGATAATGTATATGTTTCCCATATACATTATTAATGGAGTGCTCCCAATCTGT


Hap_1               -----ATACTCATATACATTCTGCTACATGGCCAGCAGCTTTTAGCATGAAACAGGAGAA
Hap_2               -----ATACTCATATACATTCTGCTACATGGCCAGCAGCTTTTAGCATGAAACAGGAGAA
Hap_3               -----ATACTCATATACATTCTGCTACATGGCCAGCAGCTTTTAGCATGAAACAGGAGAA
Hap_4               -----ATACTCATATACATTCTGCTACATGGCCAGCAGCTTTTAGCATGAAACAGGCGAA
Hap_5               -----ATACTCATATACATTCTGCTACATGGCCAGCAGCTTTTAGCATGAAACAGGCGAA
Hap_6               -----ATACTCATATACATTCTGCTACATGGCCAGCAGCTTTTAGCATGAAACAGGCGAA
Hap_7               -----ATACTCATATACATTCTGCTACATGGCCAGCAGCTTTTAGCATGAAACAGGCGAA
Hap_8               TAAATATACTCATATACATTCTGCTACATGGCCAGCAGCTTTTAGCATGAAACAGGCGAA
Hap_9               TAAATATACTCATATACATTCTGCTACATGGCCAGCAGCTTTTAGCATGAAACAGGCGAA
```

```
Hap_10          TAAATATACTCATATACATTCTGCTACATGGCCAGCAGCTTTTAGCATGAAACAGGCGAA
Hap_11          TAAATATACTCATATACATTCTGCTACATGGCCAGCAGCTTTTAGCATGAAACAGGCGAA
Hap_12          TAAATATACTCATATACATTCTGCTACATGGCCAGCAGCTTTTAGCATGAAACAGGCGAA
Hap_13          TAAATATACTCATATACATTCTGCTACATGGCCAGCAGCTTTTAGCATGAAACAGGCGAA
Hap_14          TAAATATACTCATATACATTCTGCTACATGGCCAGCAGCTTTTAGCATGAAACAGGCGAA
Hap_15          TAAATATACTCATATACATTCTGCTACATGGCCAGCAGCTTTTAGCATGAAACAGGCGAA
Hap_16          TAAATATACTCATATACATTCTGCTACATGGCCAGCAGCTTTTAGCATGAAACAGGCGAA
Hap_17          TAAATATACTCATATACATTCTGCTACATGGCCAGCAGCTTTTAGCATGAAACAGGCGAA
Hap_18          TAAATATACTCATATACATTCTGCTACATGGCCAGCAGCTTTTAGCATGAAACAGGCGAA
Hap_19          TAAATATACTCATATACATTCTGCTACATGGCCAGCAGCTTTTAGCATGAAACAGGCGAA
Hap_20          TAAATATACTCATATACATTCTGCTACATGGCCAGCAGCTTTTAGCATGAAACAGGCGAA
Hap_21          TAAATATACTCATATACATTCTGCTACATGGCCAGCAGCTTTTAGCATGAAACAGGCGAA
                     *************************************************** ***

Hap_1           GCAGAGCACAAACCTGTCTTCTTAAACGACGAAGTGTCTTCACCAATAGGTCAGGATTCC
Hap_2           GCAGAGCACAAACCTGTCTTCTTAAACGACGAAGTGTCTTCACCAATAGGTCAGGATTCC
Hap_3           GCAGAGCACAAACCTGTCTTCTTAAACGACGAAGTGTCTTCACCAATAGGTCAGGATTCC
Hap_4           GCAGAGCACAAACCTGTCTTCTTAAACGACGAAGTGTCTTCACCAATAGGTCAGGATTCC
Hap_5           GCAGAGCACAAACCTGTCTTCTTAAACGACGAAGTGTCTTCACCAATAGGTCAGGATTCC
Hap_6           GCAGAGCACAAACCTGTCTTCTTAAACGACGAAGTGTCTTCACCAATAGGTCAGGATTCC
Hap_7           GCAGAGCACAAACCTGTCTTCTTAAACGACGAAGTGTCTTCACCAATAGGTCAGGATTCC
Hap_8           GCAGAGCACAAACCTGTCTTCTTAAACGACGAAGTGTCTTCACCAATAGGTCAGGATTCC
Hap_9           GCAGAGCACAAACCTGTCTTCTTAAACGACGAAGTGTCTTCACCAATAGGTCAGGATTCC
Hap_10          GCAGAGCACAAACCTGTCTTCTTAAACGACGAAGTGTCTTCACCAATAGGTCAGGATTCC
Hap_11          GCAGAGCACAAACCTGTCTTCTTAAACGACGAAGTGTCTTCACCAATAGGTCAGGATTCC
Hap_12          GCAGAGCACAAACCTGTCTTCTTAAACGACGAAGTGTCTTCACCAATAGGTCAGGATTCC
Hap_13          GCAGAGCACAAACCTGTCTTCTTAAACGACGAAGTGTCTTCACCAATAGGTCAGGATTCC
Hap_14          GCAGAGCACAAACCTGTCTTCTTAAACGACGAAGTGTCTTCACCAATAGGTCAGGATTCC
Hap_15          GCAGAGCACAAACCTGTCTTCTTAAACGACGAAGTGTCTTCACCAATAGGTCAGGATTCC
Hap_16          GCAGAGCACAAACCTGTCTTCTTAAACGACGAAGTGTCTTCACCAATAGGTCAGGATTCC
Hap_17          GCAGAGCACAAACCTGTCTTCTTAAACGACGAAGTGTCTTCACCAATAGGTCAGGATTCC
Hap_18          GCAGAGCACAAACCTGTCTTCTTAAACGACGAAGTGTCTTCACCAATAGGTCAGGATTCC
Hap_19          GCAGAGCACAAACCTGTCTTCTTAAACGACGAAGTGTCTTCACCAATAGGTCAGGATTCC
Hap_20          GCAGAGCACAAACCTGTCTTCTTAAACGACGAAGTGTCTTCACCAATAGGTCAGGATTCC
Hap_21          GCAGAGCACAAACCTGTCTTCTTAAACGACGAAGTGTCTTCACCAATAGGTCAGGATTCC
                ************************************************************

Hap_1           TATGAATTCCAACCCAACAACCATTGACAAATATTTTAGCAGCTTGAGGAATGACTGCCG
Hap_2           TATGAATTCCAACCCAACAACCATTGACAAATATTTTAGCAGCTTGAGGAATGACTGCCG
Hap_3           TATGAATTCCAACCCAACAACCATTGACAAATATTTTAGCAGCTTGAGGAATGACTGCCG
Hap_4           TATGAATTCCAACCCAACAACCATTGACAAATATTTTAGCAGCTTGAGGAATGACTGCCG
Hap_5           TATGAATTCCAACCCAACAACCATTGACAAATATTTTAGCAGCTTGAGGAATGACTGCCG
Hap_6           TATGAATTCCAACCCAACAACCATTGACAAATATTTTAGCAGCTTGAGGAATGACCGCCG
Hap_7           TATGAATTCCAACCCAACAACCATTGACAAATATTTTAGCAGCTTGAGGAATGACTGCCG
Hap_8           TATGAATTCCAACCCAACAACCATTGACAAATATTTTAGCAGCTTGAGGAATGACTGCCG
Hap_9           TATGAATTCCAACCCAACAACCATTGACAAATATTTTAGCAGCTTGAGGAATGACTGCCG
Hap_10          TATGAATTCCAACCCAACAACCATTGACAAATATTTTAGCAGCTTGAGGAATGACTGCCG
Hap_11          TATGAATTCCAACCCAACAACCATTGACAAATATTTTAGCAGCTTGAGGAATGACTGCCG
Hap_12          TATGAATTCCAACCCAACAACCATTGACAAATATTTTAGCAGCTTGAGGAATGACTGCCG
Hap_13          TATGAATTCCAACCCAACAACCATTGACAAATATTTTAGCAGCTTGAGGAATGACTGCCG
Hap_14          TATGAATTCCAACCCAACAACCATTGACAAATATTTTAGCAGCTTGAGGAATGACTGCCG
```

Hap_15 TATGAATTCCAACCCAACAACCATTGACAAATATTTTAGCAGCTTGAGGAATGACTGCCG
Hap_16 TATGAATTCCAACCCAACAACCATTGACAAATATTTTAGCAGCTTGAGGAATGACTGCCG
Hap_17 TATGAATTCCAACCCAACAACCATTGACAAATATTTTAGCAGCTTGAGGAATGACTGCCG
Hap_18 TATGAATTCCAACCCAACAACCATTGACAAATATTTTAGCAGCTTGAGGAATGACTGCCG
Hap_19 TATGAATTCCAACCCAACAACCATTGACAAATATTTTAGCAGCTTGAGGAATGACTGCCG
Hap_20 TATGAATTCCAACCCAACAACCATTGACAAATATTTTAGCAGCTTGAGGAATGACTGCCG
Hap_21 TATGAATTCCAACCCAACAACCATTGACAAATATTTTAGCAGCTTGAGGAATGACTGCCG
*** ****

Hap_1 GTGATATCTCCTGTTAAAAATGAATCCAGATCGAAGTCAGTGACTGTTGTAATTGGTAAA
Hap_2 GTGATATCTCCTGTTAAAAATGAATCCAGATCGAAGTCAGTGACTGTTGTAATTGGTAAA
Hap_3 GTGATATCTCCTGTTAAAAATGAATCCAGATCGAAGTCAGTGACTGTTGTAATTGGTAAA
Hap_4 GTGATATCTCCTGTTAAAAATGAATCCAGATCGAAGTCAGTGACTGTTGTAATTGGTAAA
Hap_5 GTGATATCTCCTGTTAAAAATGAATCCAGATCGAAGTCAGTGACTGTTGTAATTGGTAAA
Hap_6 GTGATATCTCCTGTTAAAAATGAATCCAGATCGAAGTCAGTGACTGTTGTAATTGGTAAA
Hap_7 GTGATATCTCCTATTAAAAATGAATCCAGATCGAAGTCAGTGACTGTTGTAATTGGTAAA
Hap_8 GTGATATCTCCTGTTAAAAATGAATCCAGATCGAAGTCAGTGACTGTTGTAATTGGTAAA
Hap_9 GTGATATCTCCTGTTAAAAATGAATCCAGATCGAAGTCAGTGACTGTTGTAATTGGTAAA
Hap_10 GTGATATCTCCTGTTAAAAATGAATCCAGATCGAAGTCAGTGACTGTTGTAATTGGTAAA
Hap_11 GTGATATCTCCTGTTAAAAATGAATCCAGATCGAAGTCAGTGACTGTTGTAATTGGTAAA
Hap_12 GTGATATCTCCTGTTAAAAATGAATCCAGATCGAAGTCAGTGACTGTTGTAATTGGTAAA
Hap_13 GTGATATCTCCTGTTAAAAATGAATCCAGATCGAAGTCAGTGACTGTTGTAATTGGTAAA
Hap_14 GTGATATCTCCTGTTAAAAATGAATCCAGATCGAAGTCAGTGACTGTTGTAATTGGTAAA
Hap_15 GTGATATCTCCTGTTAAAAATGAATCCAGATCGAAGTCAGTGACTGTTGTAATTGGTAAA
Hap_16 GTGATATCTCCTGTTAAAAATGAATCCAGATCGAAGTCAGTGACTGTTGTAATTGGTAAA
Hap_17 GTGATATCTCCTGTTAAAAATGAATCCAGATCGAAGTCAGTGACTGTTGTAATTGGTAAA
Hap_18 GTGATATCTCCTGTTAAAAATGAATCCAGATCGAAATCAGTGACTGTTGTAATTGGTAAA
Hap_19 GTGATATCTCCTGTTAAAAATGAATCCAGATCGAAGTCAGTGACTGTTGTAATTGGTAAA
Hap_20 GTGATATCTCCTGTTAAAAATGAATCCAGATCGAAGTCAGTGACTGTTGTAATTGGTAAA
Hap_21 GTGATATCTCCTGTTAAAAATGAATCCAGATCGAAGTCAGTGACTGTTGTAATTGGTAAA
************ ********************** ************************

Hap_1 TGACACAAAACTTAAATCTTGGTAAGATAAGCAAATGATAATGCATTCGCACACATTGAG
Hap_2 TGACACAAAACTTAAATCTTGGTAAGATAAGCAAATGATAATGCATTCGCACACATTGAG
Hap_3 TGACACAAAACTTAAATCTTGGTAAGATAAGCAAATGATAATGCATTCGCACACATTGAG
Hap_4 TGACACAAAACTTAAATCTTGGTAAGATAAGCAAATGATAATGCATTCGCACACATTGAG
Hap_5 TGACACAAAACTTAAATCTTGGTAAGATAAGCAAATGATAATGCATTCGCACACATTGAG
Hap_6 TGACACAAAACTTAAATCTTGGTAAGATAAGCAAATGATAATGCATTCGCACACATTGAG
Hap_7 TGACACAAAACTTAAATCTTGGTAAGATAAGCAAATGATAATGCATTCGCACACATTGAG
Hap_8 TGACACAAAACTTAAATCTTGGTAAGATAAGCAAATGATAATGCATTCGCACACATTGAG
Hap_9 TGACACAAAACTTAAATCTTGGTAAGATAAGCAAATGATAATGCATTCGCACACATTGAG
Hap_10 TGACACAAAACTTAAATCTTGGTAAGATAAGCAAATGATAATGCATTCGCACACATTGAG
Hap_11 TGACACAAAACTTAAATCTTGGTAAGATAAGCAAATGATAATGCATTCGCACACATTGAG
Hap_12 TGACACAAAACTTAAATCTTGGTAAGATAAGCAAATGATAATGCATTCGCACACATTGAG
Hap_13 TGACACAAAACTTAAATCTTGGTAAGATAAGCAAATGATAATGCATTCGCACACATTGAG
Hap_14 TGACACAAAACTTAAATCTTGGTAAGATAAGCAAATGATAATGCATTCGCACACATTGAG
Hap_15 TGACACAAAACATAAATCTTGGTAAGATAAGCAAATGATAATGCATTCGCACACATTGAG
Hap_16 TGACACAAAACTTAAATCTTGGTAAGATAAGCAAATGATAATGCATTCGCACACATTGAG
Hap_17 TGACACAAAACTTAAATCTTGGTAAGATAAGCAAATGATAATGCATTCGCACACATTGAG
Hap_18 TGACACAAAACTTAAATCTTGGTAAGATAAGCAAATGATAATGCATTCGCACACATTGAG
Hap_19 TGACACAAAACTTAAATCTTGGTAAGATAAGCAAATGATAATGCATTCGCACACATTGAG

```
Hap_20    TGACACAAAACTTAAATCTTGGTAAGATAAGCAAATGATAATGCATTCGCACACATTGAG
Hap_21    TGACACAAAACTCAAATCTTGGTAAGATAAGCAAATGATAATGCATTCGCACACATTGAG
          *********** ************************************************

Hap_1     AAGGGGATAAAACAAACCTCAAAATTTTCTGTGCCCCATTCTTCCAAAAACTCCAAAATA
Hap_2     AAGGGGATAAAACAAACCTCAAAATTTTCTGTGCCCCATTCTTCCAAAAATTCCAAAATA
Hap_3     AAGGGGATAAAACAAACCTCAAAATTTTCTGTGCCCCATTCTTCCAAAAATTCCAAAATA
Hap_4     AAGGGGATAAAACAAACCTCAAAATTTTCTGTGCCCCATTCTTCCAAAAATTCCAAAATA
Hap_5     AAGGGGATAAAACAAACCTCAAAATTTTCTGTGCCCCATTCTTCCAAAAATTCCAAAATA
Hap_6     AAGGGGATAAAACAAACCTCAAAATTTTCTGTGCCCCATTCTTCCAAAAATTCCAAAATA
Hap_7     AAGGGGATAAAACAAACCTCAAAATTTTCTGTGCCCCATTCTTCCAAAAATTCCAAAATA
Hap_8     AAGGGGATAAAACAAACCTCAAAATTTTCTGTGCCCCATTCTTCCAAAAATTCCAAAATA
Hap_9     AAGGGGATAAAACAAACCTCAAAATTTTCTGTGCCCCATTCTTCCAAAAATTCCAAAATA
Hap_10    AAGGGGATAAAACAAACCTCAAAATTTTCTGTGCCCCATTCTTCCAAAAATTCCAAAATA
Hap_11    AAGGGGATAAAACAAACCTCAAAATTTTCTGTGCCCCATTCTTCCAAAAATTCCAAAATA
Hap_12    AAGGGGATAAAACAAACCTCAAAATTTTCTGTGCCCCATTCTTCCAAAAATTCCAAAATA
Hap_13    AAGGGGATAAAACAAACCTCAAAATTTTCTGTGCCCCATTCTTCCAAAAATTCCAAAATA
Hap_14    AAGGGGATAAAACAAACCTCAAAATTTTCTGTGCCCCATTCTTCCAAAAATTCCAAAATA
Hap_15    AAGGGGATAAAACAAACCTCAAAATTTTCTGTGCCCCATTCTTCCAAAAATTCCAAAATA
Hap_16    AAGGGGATAAAACAAACCTCAAAATTTTCTGTGCCCCATTCTTCCAAAAATTCCAAAATA
Hap_17    AAGGGGATAAAACAAACCTCAAAATTTTCTGTGCCCCATTCTTCCAAAAATTCCAAAATA
Hap_18    A------TAAAACAAACCTCAAAATTTTCTGTGCCCCATTCTTCCAAAAATTCCAAAATA
Hap_19    A------TAAAACAAACCTCAAAATTTTCTGTGCCCCATTCTTCCAAAAATTCCAAAATA
Hap_20    A------TAAAACAAACCTCAAAATTTTCTGTGCCCCATTCTTCCAAAAATTCCAAAATA
Hap_21    AAGGGGATAAAACAAACCTCAAAATTTTCTGTGCCCCATTCTTCCAAAAATTCCAAAATA
          *      ******************************************* *********

Hap_1     GGATTTGCGGCAGAGCCAACAGTGATATAAACCATCAAGGCAAGATTTTTAACCAAGCCA
Hap_2     GGATTTGCGGCAGAGCCAACAGTGATATAAACCATCAAGGCAAGATTTTTAACCAAGCCA
Hap_3     GGATTTGCGGCAGAGCCAACAGTGATATAAACCATCAAGGCAAGATTTTTAACCAAGCCA
Hap_4     GGATTTGCGGCAGAGCCAACAGTGATATAAACCATCAAGGCAAGATTTTTAACCAAGCCA
Hap_5     GGATTTGCGGCAGAGCCAACAGTGATATAAACCATCAAGGCAAGATTTTTAACCAAGCCA
Hap_6     GGATTTGCGGCAGAGCCAACAGTGATATAAACCATCAAGGCAAGATTTTTAACCAAGCCA
Hap_7     GGATTTGCGGCAGAGCCAACAGTGATATAAACCATCAAGGCAAGATTTTTAACCAAGCCA
Hap_8     GGATTTGCGGCAGAGCCAACAGTGATATAAACCATCAAGGCAAGATTTTTAACCAAGCCA
Hap_9     GGATTTGCGGCAGAGCCAACAGTGATATAAACCATCAAGGCAAGATTTTTAACCAAGCCA
Hap_10    GGATTTGCGGCAGAGCCAACAGTGATATAAACCATCAAGGCAAGATTTTTAACCAAGCCA
Hap_11    GGATTTGCGGCAGAGCCAACAGTGATATAAACCATCAAGGCAAGATTTTTAACCAAGCCA
Hap_12    GGATTTGCGGCAGAGCCAACAGTGATATAAACCATCAAGGCAAGATTTTTAACCAAGCCA
Hap_13    GGATTTGCGGCAGAGCCAACAGTGATATAAACCATCAAGGCAAGATTTTTAACCAAGCCA
Hap_14    GGATTTGCGGCAGAGCCAACAGTGATATAAACCATCAAGGCAAGATTTTTAACCAAGCCA
Hap_15    GGATTTGCGGCAGAGCCAACAGTGATATAAACCATCAAGGCAAGATTTTTAACCAAGCCA
Hap_16    GGATTTGCGGCAGAGCCAACAGTGATATAAACCATCAAGGCAAGATTTTTAACCAAGCCA
Hap_17    GGATTTGCGGCAGAGCCAACAGTGATATAAACCATCAAGGCAAGATTTTTAACCAAGCCA
Hap_18    GGATTTGCGGCAGAGCCAACAGTGATATAAACCATCAAGGCAAGATTTTTAACCAAGCCA
Hap_19    GGATTTGCGGCAGAGCCAACAGTGATATAAACCATCAAGGCAAGATTTTTAACCAAGCCA
Hap_20    GGATTTGCGGCAGAGCCAACAGTGATATAAACCATCAAGGCAAGATTTTTAACCAAGCCA
Hap_21    GGATTTGCGGCAGAGCCAACAGTGATATAAACCATCAAGGCAAGATTTTTAACCAAGCCA
          ************************************************************

Hap_1     CAAGCCTGAATAAAGGTGTAGAAAACAGAAACCATTGACCAACTCATTTGACAAAGAAAA
```

```
Hap_2     CAAGCCTGAATAAAGGTGTAGAAAACAGAAACCATTGACCAACTCATTTGACAAAGAAAA
Hap_3     CAAGCCTGAATAAAGGTGTAGAAAACAGAAACCATTGACCAACTCATTTGACAAAGAAAA
Hap_4     CAAGCCTGAATAAAGGTGTAGAAAACAGAAACCATTGACCAACTCATTTGACAAAGAAAA
Hap_5     CAAGCCTGAATAAAGGTGTAGAAAACAGAAACCATTGACCAACTCATTTGACAAAGAAAA
Hap_6     CAAGCCTGAATAAAGGTGTAGAAAACAGAAACCATTGACCAACTCATTTGACAAAGAAAA
Hap_7     CAAGCCTGAATAAAGGTGTAGAAAACAGAAACCATTGACCAACTCATTTGACAAAGAAAA
Hap_8     CAAGCCTGAATAAAGGTGTAGAAAACAGAAACCATTGACCAACTCATTTCACAAAGAAAA
Hap_9     CAAGCCTGAATAAAGGTGTAGAAAACAGAAACCATTGACCAACTCATTTCACAAAGAAAA
Hap_10    CAAGCCTGAATAAAGGTGTAGAAAACAGAAACCATTGACCAACTCATTTCACAAAGAAAA
Hap_11    CAAGCCTGAATAAAGGTGTAGAAAACAGAAACCATTGACCAACTCATTTCACAAAGAAAA
Hap_12    CAAGCCTGAATAAAGGTGTAGAAAACAGAAACCATTGACCAACTCATTTCACAAAGAAAA
Hap_13    CAAGCCTGAATAAAGGTGTAGAAAACAGAAACCATTGACCAACTCATTTCACAAAGAAAA
Hap_14    CAAGCCTGAATAAAGGTGTAGAAAACAGAAACCATTGACCAACTCATTTCACAAAGAAAA
Hap_15    CAAGCCTGAATAAAGGTGTAGAAAACAGAAACCATTGACCAACTCATTTCACAAAGAAAA
Hap_16    CAAGCCTGAATAAAGGTGTAGAAAACAGAAACCATTGGCCAACTCATTTCACAAAGAAAA
Hap_17    CAAGCCTGAATAAAGGTGTAGAAAACAGAAACCATTGACCAACTCATTTCACAAAGAAAA
Hap_18    CAAGCCTGAATAAAGGTGTAGAAAACAGAAACCATTGACCAACTCATTTCACAAAGAAAA
Hap_19    CAAGCCTGAATAAAGGTGTAGAAAACAGAAACCATTGACCAACTCATTTCACAAAGAAAA
Hap_20    CAAGCCTGAATAAAGGTGTAGAAAACAGAAACCATTGACCAACTCATTTCACAAAGAAAA
Hap_21    CAAGCCTGAATAAAGGTGTAGAAAACAGAAACCATTGACCAACTCATTTCACAAAGAAAA
          ************************************* *********** **********

Hap_1     TTTTAGAATGTGAATAAC
Hap_2     TTTTAGAATGTGAATAAC
Hap_3     TTTTAGAATGTGAATAAC
Hap_4     TTTTAGAATGTGAATAAC
Hap_5     TTTTAGAATGTGAATAAC
Hap_6     TTTTAGAATGTGAATAAC
Hap_7     TTTTAGAATGTGAATAAC
Hap_8     TTTTAGAATGTGAATAAC
Hap_9     TTTTAGAATGTGAATAAC
Hap_10    TTTTAGAATGTGAATAAC
Hap_11    TTTTAGAATGTGAATAAC
Hap_12    TTTTAGAATGTGAATAAC
Hap_13    TTTTAGAATGTGAATAAC
Hap_14    TTTTAGAATGTGAATAAC
Hap_15    TTTTAGAATGTGAATAAC
Hap_16    TTTTAGAATGTGAATAAC
Hap_17    TTTTAGAATGTGAATAAC
Hap_18    TTTTAGAATGTGAATAAC
Hap_19    TTTTAGAATGTGAATAAC
Hap_20    TTTTAGAATGTGAATAAC
Hap_21    TTTTAGAATGTGAATAAC
          ******************
```

Supplementary Figure S3. Phylogenetic tree (neighbor-joining) of 88 wild barley accessions based on the *RPB2* gene. Each accession is denoted with different colored squares: Tibet (Wb-T, red), Southwest Asia (Wb-S, purple), and Central Asia (Wb-C, orange), respectively.

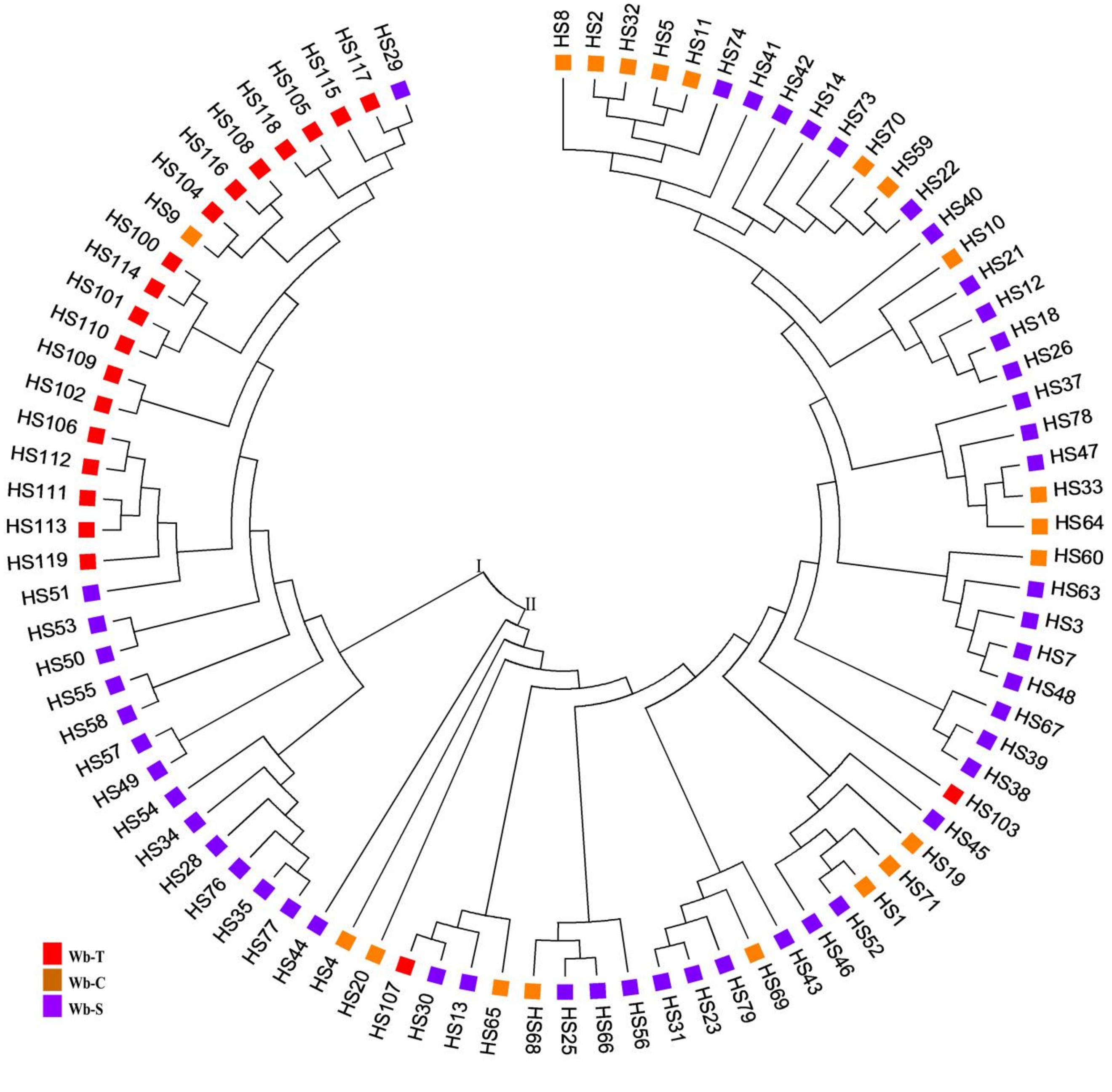

Supplementary Figure S4. Analysis of population structure of 88 wild barley accessions using STRUCTURE. (A) Estimated LnP(*K*) of possible clusters (*K*) from 1 to 10. (B) Δ*K* based on rate of change of LnP (*K*) between successive *K* values. (C) Population structure based on *K* = 2. Red represents Subgroup Q1; green, Subgroup Q2. Each part of figure was manually combined using Adobe Photoshop 9.0 (Adobe Systems Inc., San Jose, CA, USA).

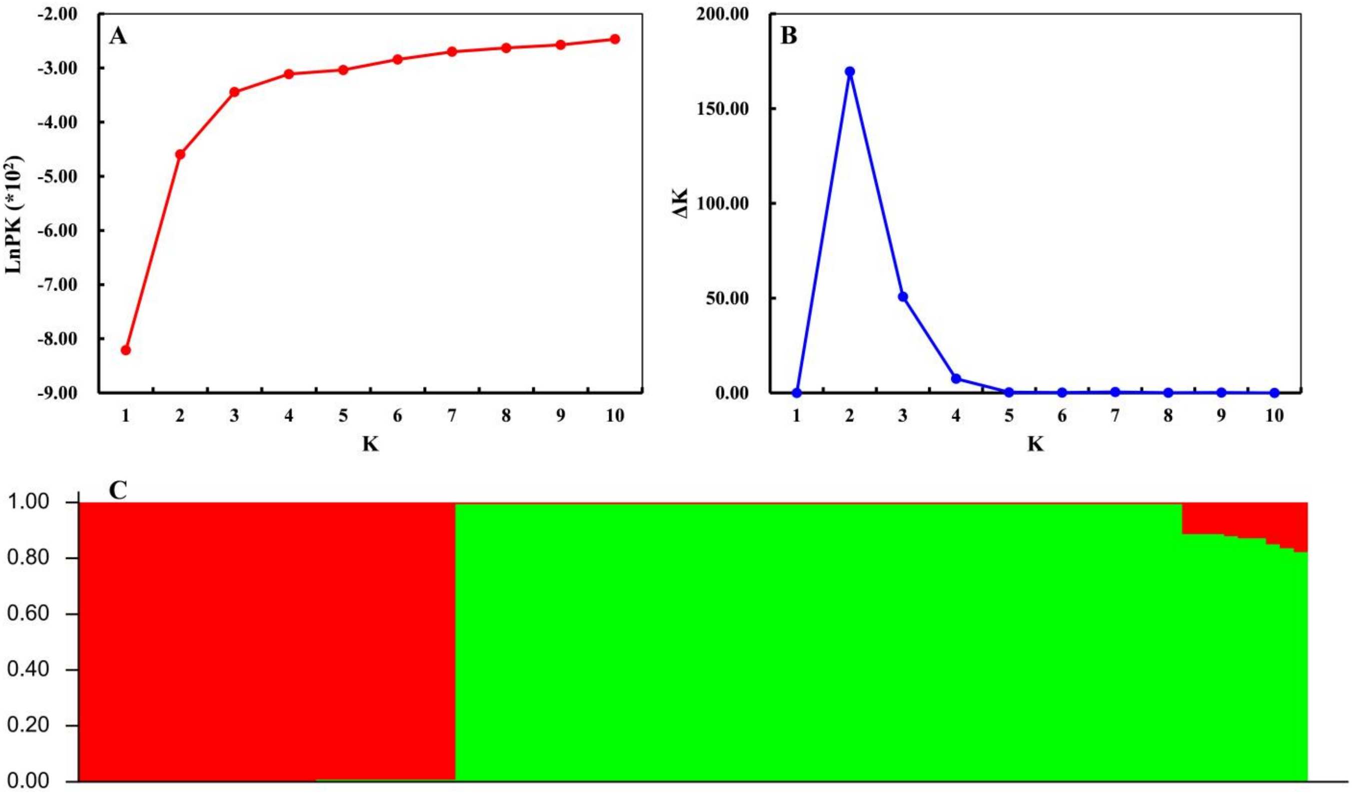

Supplementary Table S1. Result of STRUCTURE analysis and inferred ancestry of individuals in 88 wild barley accessions. (A) The Evanno table output by STRUCTURE HARVESTER[85]. (B) The raw STRUCTURE output by STRUCTURE HARVESTER[85]. (C) Inferred ancestry of individuals based on 88 wild accessions.

(A) The Evanno table output by STRUCTURE HARVESTER[85].

K	Reps	Mean LnP(K)	Stdev LnP(K)	Ln'(K)	|Ln''(K)|	Delta K
1	20	-821.060000	0.059824	—	—	—
2	20	-459.530000	1.453164	361.530000	246.500000	169.629912
3	20	-344.500000	1.610819	115.030000	81.765000	50.759903
4	20	-311.235000	3.402982	33.265000	25.610000	7.525751
5	20	-303.580000	32.051170	7.655000	11.580000	0.361297
6	20	-284.345000	20.495660	19.235000	4.870000	0.237611
7	20	-269.980000	14.960249	14.365000	7.290000	0.487291
8	20	-262.905000	15.811105	7.075000	1.395000	0.088229
9	20	-257.225000	25.034689	5.680000	4.885000	0.195129
10	20	-246.660000	24.501201	10.565000	—	—

(B) The raw STRUCTURE output by STRUCTURE HARVESTER[85].

File name	Run #	K	Est. Ln prob. of data	Mean value of Ln likelihood	Variance of Ln likelihood
ResultsAW-2_run_5_f	5	1	-821.1	-814.9	12.5
ResultsAW-2_run_15_f	15	1	-821.0	-814.9	12.2
ResultsAW-2_run_14_f	14	1	-821.1	-814.9	12.3
ResultsAW-2_run_13_f	13	1	-821.0	-814.9	12.2
ResultsAW-2_run_10_f	10	1	-821.0	-814.9	12.2
ResultsAW-2_run_3_f	3	1	-821.1	-814.9	12.4
ResultsAW-2_run_20_f	20	1	-821.1	-814.9	12.4
ResultsAW-2_run_17_f	17	1	-821.1	-814.9	12.4
ResultsAW-2_run_12_f	12	1	-821.1	-814.9	12.3
ResultsAW-2_run_2_f	2	1	-821.1	-814.9	12.3
ResultsAW-2_run_4_f	4	1	-821.0	-814.9	12.2
ResultsAW-2_run_6_f	6	1	-821.0	-814.9	12.2
ResultsAW-2_run_11_f	11	1	-821.1	-814.9	12.3
ResultsAW-2_run_18_f	18	1	-821.1	-814.9	12.4
ResultsAW-2_run_7_f	7	1	-821.0	-814.9	12.3

ResultsAW-2_run_1_f	1	1	-821.1	-814.9	12.3
ResultsAW-2_run_19_f	19	1	-821.0	-814.9	12.3
ResultsAW-2_run_8_f	8	1	-821.0	-814.9	12.2
ResultsAW-2_run_16_f	16	1	-821.2	-814.9	12.4
ResultsAW-2_run_9_f	9	1	-821.0	-814.9	12.3
ResultsAW-2_run_39_f	39	2	-458.2	-440.9	34.6
ResultsAW-2_run_37_f	37	2	-458.0	-441.0	33.9
ResultsAW-2_run_22_f	22	2	-459.7	-439.2	40.9
ResultsAW-2_run_28_f	28	2	-460.4	-439.5	41.8
ResultsAW-2_run_36_f	36	2	-460.1	-440.8	38.6
ResultsAW-2_run_33_f	33	2	-458.5	-438.7	39.5
ResultsAW-2_run_40_f	40	2	-461.4	-440.1	42.7
ResultsAW-2_run_38_f	38	2	-456.8	-440.1	33.5
ResultsAW-2_run_26_f	26	2	-459.8	-441.0	37.7
ResultsAW-2_run_24_f	24	2	-458.7	-440.2	37.1
ResultsAW-2_run_29_f	29	2	-461.1	-439.6	42.9
ResultsAW-2_run_21_f	21	2	-462.1	-439.4	45.5
ResultsAW-2_run_30_f	30	2	-458.0	-441.3	33.3
ResultsAW-2_run_25_f	25	2	-461.6	-439.8	43.6
ResultsAW-2_run_31_f	31	2	-460.3	-440.4	39.7
ResultsAW-2_run_27_f	27	2	-457.7	-440.6	34.4
ResultsAW-2_run_23_f	23	2	-459.3	-439.1	40.5
ResultsAW-2_run_35_f	35	2	-460.9	-440.1	41.6
ResultsAW-2_run_32_f	32	2	-459.0	-439.9	38.1
ResultsAW-2_run_34_f	34	2	-459.0	-440.9	36.3
ResultsAW-2_run_50_f	50	3	-345.0	-327.9	34.1
ResultsAW-2_run_57_f	57	3	-344.0	-328.2	31.6
ResultsAW-2_run_54_f	54	3	-343.1	-327.7	30.8
ResultsAW-2_run_55_f	55	3	-344.1	-327.9	32.4
ResultsAW-2_run_49_f	49	3	-345.0	-327.5	34.9
ResultsAW-2_run_60_f	60	3	-349.0	-328.7	40.7
ResultsAW-2_run_59_f	59	3	-345.6	-327.8	35.7
ResultsAW-2_run_51_f	51	3	-344.8	-327.9	33.8
ResultsAW-2_run_43_f	43	3	-344.1	-327.7	32.7
ResultsAW-2_run_56_f	56	3	-346.0	-328.3	35.5
ResultsAW-2_run_58_f	58	3	-343.5	-328.0	31.1
ResultsAW-2_run_44_f	44	3	-343.9	-327.5	32.7
ResultsAW-2_run_53_f	53	3	-344.2	-328.2	31.9

ResultsAW-2_run_47_f	47	3	-343.3	-327.8	31.0
ResultsAW-2_run_41_f	41	3	-343.3	-327.5	31.6
ResultsAW-2_run_45_f	45	3	-344.5	-327.7	33.7
ResultsAW-2_run_48_f	48	3	-341.7	-327.2	29.0
ResultsAW-2_run_42_f	42	3	-343.6	-327.7	31.7
ResultsAW-2_run_52_f	52	3	-343.8	-327.6	32.5
ResultsAW-2_run_46_f	46	3	-347.5	-328.2	38.5
ResultsAW-2_run_75_f	75	4	-309.1	-280.9	56.4
ResultsAW-2_run_64_f	64	4	-306.1	-279.7	52.8
ResultsAW-2_run_66_f	66	4	-311.1	-282.9	56.5
ResultsAW-2_run_65_f	65	4	-313.3	-281.8	62.9
ResultsAW-2_run_73_f	73	4	-312.9	-281.4	63.0
ResultsAW-2_run_63_f	63	4	-310.4	-280.8	59.3
ResultsAW-2_run_78_f	78	4	-315.0	-281.7	66.6
ResultsAW-2_run_80_f	80	4	-309.6	-281.9	55.3
ResultsAW-2_run_79_f	79	4	-307.5	-281.2	52.6
ResultsAW-2_run_76_f	76	4	-309.5	-281.7	55.5
ResultsAW-2_run_72_f	72	4	-312.3	-281.6	61.4
ResultsAW-2_run_61_f	61	4	-314.3	-283.4	61.7
ResultsAW-2_run_67_f	67	4	-311.3	-282.5	57.6
ResultsAW-2_run_68_f	68	4	-310.1	-282.2	55.8
ResultsAW-2_run_74_f	74	4	-312.0	-281.8	60.3
ResultsAW-2_run_77_f	77	4	-302.4	-277.3	50.2
ResultsAW-2_run_62_f	62	4	-316.9	-284.9	64.0
ResultsAW-2_run_71_f	71	4	-315.9	-282.5	66.8
ResultsAW-2_run_69_f	69	4	-312.4	-282.0	60.8
ResultsAW-2_run_70_f	70	4	-312.6	-281.6	61.9
ResultsAW-2_run_89_f	89	5	-338.9	-263.2	151.3
ResultsAW-2_run_93_f	93	5	-329.1	-271.2	115.7
ResultsAW-2_run_82_f	82	5	-360.3	-281.2	158.2
ResultsAW-2_run_84_f	84	5	-271.2	-241.2	59.9
ResultsAW-2_run_83_f	83	5	-330.0	-271.4	117.2
ResultsAW-2_run_97_f	97	5	-274.0	-240.2	67.7
ResultsAW-2_run_81_f	81	5	-274.9	-241.8	66.2
ResultsAW-2_run_88_f	88	5	-277.3	-242.4	69.8
ResultsAW-2_run_91_f	91	5	-281.7	-241.4	80.7
ResultsAW-2_run_99_f	99	5	-324.4	-268.3	112.3
ResultsAW-2_run_100_f	100	5	-278.3	-242.8	71.0

ResultsAW-2_run_90_f	90	5	-277.5	-241.2	72.7
ResultsAW-2_run_86_f	86	5	-276.3	-242.0	68.7
ResultsAW-2_run_98_f	98	5	-334.8	-274.3	121.0
ResultsAW-2_run_87_f	87	5	-277.0	-240.2	73.7
ResultsAW-2_run_94_f	94	5	-325.0	-269.1	111.6
ResultsAW-2_run_92_f	92	5	-272.3	-240.1	64.3
ResultsAW-2_run_95_f	95	5	-329.7	-271.6	116.2
ResultsAW-2_run_85_f	85	5	-358.1	-275.2	165.9
ResultsAW-2_run_96_f	96	5	-280.8	-242.5	76.6
ResultsAW-2_run_118_f	118	6	-289.0	-230.7	116.6
ResultsAW-2_run_119_f	119	6	-286.2	-231.3	109.8
ResultsAW-2_run_109_f	109	6	-277.3	-225.9	102.9
ResultsAW-2_run_103_f	103	6	-291.8	-231.1	121.4
ResultsAW-2_run_102_f	102	6	-296.8	-228.7	136.2
ResultsAW-2_run_112_f	112	6	-250.0	-215.1	69.8
ResultsAW-2_run_111_f	111	6	-293.4	-231.1	124.5
ResultsAW-2_run_101_f	101	6	-292.2	-228.9	126.7
ResultsAW-2_run_120_f	120	6	-231.9	-195.1	73.5
ResultsAW-2_run_116_f	116	6	-286.1	-229.6	113.0
ResultsAW-2_run_117_f	117	6	-283.5	-229.3	108.5
ResultsAW-2_run_106_f	106	6	-255.4	-215.8	79.2
ResultsAW-2_run_113_f	113	6	-315.6	-231.8	167.6
ResultsAW-2_run_104_f	104	6	-291.6	-229.4	124.4
ResultsAW-2_run_108_f	108	6	-295.5	-231.3	128.5
ResultsAW-2_run_115_f	115	6	-292.8	-228.8	127.9
ResultsAW-2_run_107_f	107	6	-302.8	-231.7	142.2
ResultsAW-2_run_105_f	105	6	-257.6	-216.2	82.8
ResultsAW-2_run_114_f	114	6	-305.3	-228.6	153.4
ResultsAW-2_run_110_f	110	6	-292.1	-228.6	127.1
ResultsAW-2_run_127_f	127	7	-264.4	-213.3	102.2
ResultsAW-2_run_125_f	125	7	-271.2	-205.7	131.0
ResultsAW-2_run_137_f	137	7	-284.3	-210.1	148.4
ResultsAW-2_run_139_f	139	7	-271.4	-205.9	131.1
ResultsAW-2_run_130_f	130	7	-264.7	-206.2	117.0
ResultsAW-2_run_131_f	131	7	-267.0	-201.8	130.5
ResultsAW-2_run_133_f	133	7	-245.8	-184.3	123.0
ResultsAW-2_run_138_f	138	7	-257.0	-210.5	93.1
ResultsAW-2_run_135_f	135	7	-277.7	-205.9	143.5

ResultsAW-2_run_134_f	134	7	-265.0	-203.5	123.0
ResultsAW-2_run_128_f	128	7	-267.8	-205.3	125.1
ResultsAW-2_run_122_f	122	7	-277.2	-204.6	145.1
ResultsAW-2_run_136_f	136	7	-273.1	-206.6	133.1
ResultsAW-2_run_129_f	129	7	-274.9	-204.5	140.6
ResultsAW-2_run_132_f	132	7	-261.3	-209.3	103.9
ResultsAW-2_run_124_f	124	7	-321.8	-215.9	211.9
ResultsAW-2_run_140_f	140	7	-263.2	-208.0	110.4
ResultsAW-2_run_126_f	126	7	-265.4	-205.1	120.5
ResultsAW-2_run_123_f	123	7	-255.2	-214.8	80.9
ResultsAW-2_run_121_f	121	7	-271.2	-203.9	134.5
ResultsAW-2_run_144_f	144	8	-269.3	-200.8	137.0
ResultsAW-2_run_147_f	147	8	-262.2	-198.3	127.8
ResultsAW-2_run_148_f	148	8	-229.8	-158.2	143.2
ResultsAW-2_run_141_f	141	8	-269.2	-198.0	142.4
ResultsAW-2_run_149_f	149	8	-273.4	-202.2	142.4
ResultsAW-2_run_143_f	143	8	-235.2	-160.1	150.3
ResultsAW-2_run_146_f	146	8	-267.8	-200.5	134.6
ResultsAW-2_run_157_f	157	8	-246.0	-201.1	89.9
ResultsAW-2_run_159_f	159	8	-277.0	-199.6	154.8
ResultsAW-2_run_142_f	142	8	-269.9	-200.5	138.9
ResultsAW-2_run_160_f	160	8	-273.9	-201.9	144.1
ResultsAW-2_run_150_f	150	8	-262.4	-202.3	120.2
ResultsAW-2_run_155_f	155	8	-251.6	-202.5	98.1
ResultsAW-2_run_154_f	154	8	-286.9	-199.3	175.3
ResultsAW-2_run_158_f	158	8	-247.3	-200.8	93.0
ResultsAW-2_run_156_f	156	8	-276.9	-197.7	158.2
ResultsAW-2_run_153_f	153	8	-245.5	-201.5	88.0
ResultsAW-2_run_151_f	151	8	-257.3	-201.0	112.6
ResultsAW-2_run_145_f	145	8	-272.4	-200.4	143.9
ResultsAW-2_run_152_f	152	8	-284.1	-199.6	168.9
ResultsAW-2_run_172_f	172	9	-298.9	-195.2	207.6
ResultsAW-2_run_169_f	169	9	-255.9	-195.3	121.3
ResultsAW-2_run_173_f	173	9	-253.1	-196.1	114.0
ResultsAW-2_run_162_f	162	9	-273.4	-195.6	155.5
ResultsAW-2_run_161_f	161	9	-255.5	-157.1	197.0
ResultsAW-2_run_174_f	174	9	-209.6	-156.5	106.1
ResultsAW-2_run_165_f	165	9	-303.6	-198.6	210.1

ResultsAW-2_run_175_f	175	9	-269.6	-192.8	153.6
ResultsAW-2_run_170_f	170	9	-281.9	-198.4	167.0
ResultsAW-2_run_167_f	167	9	-261.4	-195.5	131.7
ResultsAW-2_run_163_f	163	9	-262.3	-194.4	135.8
ResultsAW-2_run_179_f	179	9	-256.6	-196.6	119.9
ResultsAW-2_run_177_f	177	9	-242.9	-194.9	96.0
ResultsAW-2_run_164_f	164	9	-247.6	-194.4	106.6
ResultsAW-2_run_180_f	180	9	-276.1	-195.3	161.7
ResultsAW-2_run_171_f	171	9	-265.1	-194.9	140.2
ResultsAW-2_run_166_f	166	9	-250.2	-194.3	111.7
ResultsAW-2_run_168_f	168	9	-201.7	-157.7	87.9
ResultsAW-2_run_176_f	176	9	-249.3	-193.2	112.1
ResultsAW-2_run_178_f	178	9	-229.8	-155.9	147.8
ResultsAW-2_run_191_f	191	10	-255.6	-191.2	128.8
ResultsAW-2_run_195_f	195	10	-261.4	-193.0	136.7
ResultsAW-2_run_196_f	196	10	-260.2	-191.7	137.0
ResultsAW-2_run_181_f	181	10	-210.5	-153.0	115.1
ResultsAW-2_run_188_f	188	10	-263.1	-191.2	143.9
ResultsAW-2_run_182_f	182	10	-260.0	-190.1	139.8
ResultsAW-2_run_187_f	187	10	-237.3	-152.1	170.4
ResultsAW-2_run_186_f	186	10	-263.6	-191.7	143.9
ResultsAW-2_run_200_f	200	10	-258.7	-190.2	137.1
ResultsAW-2_run_194_f	194	10	-266.1	-193.3	145.6
ResultsAW-2_run_198_f	198	10	-260.6	-193.3	134.7
ResultsAW-2_run_193_f	193	10	-267.6	-191.9	151.3
ResultsAW-2_run_184_f	184	10	-209.4	-150.0	118.8
ResultsAW-2_run_197_f	197	10	-238.8	-151.4	174.7
ResultsAW-2_run_185_f	185	10	-203.7	-150.4	106.5
ResultsAW-2_run_199_f	199	10	-268.3	-197.3	142.0
ResultsAW-2_run_192_f	192	10	-211.9	-150.8	122.2
ResultsAW-2_run_190_f	190	10	-262.3	-190.4	143.7
ResultsAW-2_run_189_f	189	10	-203.6	-151.9	103.5
ResultsAW-2_run_183_f	183	10	-270.5	-193.4	154.2

(C) Inferred ancestry of individuals based on 88 wild accessions.

Serial number	Accession number this paper	Inferred clusters	Serial number	Accession number this paper	Inferred clusters

		Q1	Q2			Q1	Q2
1	HS1	0.006	0.994	45	HS37	0.126	0.874
2	HS10	0.005	0.995	46	HS38	0.004	0.996
3	HS100	0.995	0.005	47	HS39	0.006	0.994
4	HS101	0.994	0.006	48	HS4	0.005	0.995
5	HS102	0.993	0.007	49	HS40	0.005	0.995
6	HS103	0.006	0.994	50	HS41	0.005	0.995
7	HS104	0.995	0.005	51	HS42	0.007	0.993
8	HS105	0.994	0.006	52	HS43	0.007	0.993
9	HS106	0.993	0.007	53	HS44	0.005	0.995
10	HS107	0.005	0.995	54	HS45	0.006	0.994
11	HS108	0.994	0.006	55	HS46	0.005	0.995
12	HS109	0.994	0.006	56	HS47	0.005	0.995
13	HS11	0.004	0.996	57	HS48	0.006	0.994
14	HS110	0.993	0.007	58	HS49	0.99	0.01
15	HS111	0.992	0.008	59	HS5	0.005	0.995
16	HS112	0.993	0.007	60	HS50	0.987	0.013
17	HS113	0.993	0.007	61	HS51	0.987	0.013
18	HS114	0.994	0.006	62	HS52	0.112	0.888
19	HS115	0.993	0.007	63	HS53	0.988	0.012
20	HS116	0.993	0.007	64	HS54	0.165	0.835
21	HS117	0.993	0.007	65	HS55	0.987	0.013
22	HS118	0.994	0.006	66	HS56	0.006	0.994
23	HS119	0.992	0.008	67	HS57	0.988	0.012
24	HS12	0.005	0.995	68	HS58	0.989	0.011
25	HS13	0.006	0.994	69	HS59	0.006	0.994
26	HS14	0.006	0.994	70	HS60	0.006	0.994
27	HS18	0.005	0.995	71	HS63	0.006	0.994
28	HS19	0.004	0.996	72	HS64	0.006	0.994
29	HS2	0.005	0.995	73	HS65	0.005	0.995
30	HS20	0.005	0.995	74	HS66	0.007	0.993
31	HS21	0.006	0.994	75	HS67	0.006	0.994
32	HS22	0.005	0.995	76	HS68	0.005	0.995
33	HS23	0.003	0.997	77	HS69	0.004	0.996
34	HS25	0.005	0.995	78	HS7	0.004	0.996
35	HS26	0.004	0.996	79	HS70	0.006	0.994
36	HS28	0.123	0.877	80	HS71	0.005	0.995
37	HS29	0.993	0.007	81	HS73	0.005	0.995
38	HS3	0.005	0.995	82	HS74	0.005	0.995
39	HS30	0.004	0.996	83	HS76	0.11	0.89
40	HS31	0.003	0.997	84	HS77	0.111	0.889
41	HS32	0.005	0.995	85	HS78	0.006	0.994
42	HS33	0.135	0.865	86	HS79	0.006	0.994
43	HS34	0.165	0.835	87	HS8	0.005	0.995
44	HS35	0.107	0.893	88	HS9	0.993	0.007

Supplementary Table S2. Result of STRUCTURE analysis and inferred ancestry

of individuals in 212 total barley accessions. (A) The Evanno table output by STRUCTURE HARVESTER[85]. (B) The raw STRUCTURE output by STRUCTURE HARVESTER[85]. (C) Inferred ancestry of individuals based on 212 total barley accessions.

(A) The Evanno table output by STRUCTURE HARVESTER[85].

K	Reps	Mean LnP(K)	Stdev LnP(K)	Ln'(K)	|Ln''(K)|	Delta K
1	20	-1868.435000	0.058714	—	—	—
2	20	-893.170000	3.278173	975.265000	752.895000	229.669057
3	20	-670.800000	83.223377	222.370000	123.980000	1.489726
4	20	-572.410000	153.286323	98.390000	6.495000	0.042372
5	20	-467.525000	72.188160	104.885000	67.045000	0.928753
6	20	-429.685000	58.683462	37.840000	20.830000	0.354955
7	20	-371.015000	49.428708	58.670000	38.945000	0.787902
8	20	-351.290000	59.726719	19.725000	17.690000	0.296182
9	20	-349.255000	62.792360	2.035000	10.450000	0.166422
10	20	-336.770000	46.332575	12.485000	—	—

(B) The raw STRUCTURE output by STRUCTURE HARVESTER[85].

File name	Run #	K	Est. Ln prob. of data	Mean value of Ln likelihood	Variance of Ln likelihood
ResultsAAA-Total_run_2_f	2	1	-1868.4	-1862.3	12.4
ResultsAAA-Total_run_10_f	10	1	-1868.4	-1862.3	12.2
ResultsAAA-Total_run_1_f	1	1	-1868.4	-1862.2	12.3
ResultsAAA-Total_run_9_f	9	1	-1868.4	-1862.2	12.3
ResultsAAA-Total_run_15_f	15	1	-1868.4	-1862.2	12.3
ResultsAAA-Total_run_12_f	12	1	-1868.4	-1862.2	12.4
ResultsAAA-Total_run_19_f	19	1	-1868.4	-1862.3	12.3
ResultsAAA-Total_run_4_f	4	1	-1868.5	-1862.3	12.4
ResultsAAA-Total_run_6_f	6	1	-1868.4	-1862.3	12.2
ResultsAAA-Total_run_7_f	7	1	-1868.4	-1862.2	12.3
ResultsAAA-Total_run_3_f	3	1	-1868.4	-1862.2	12.4
ResultsAAA-Total_run_8_f	8	1	-1868.5	-1862.2	12.4
ResultsAAA-Total_run_13_f	13	1	-1868.5	-1862.3	12.4
ResultsAAA-Total_run_20_f	20	1	-1868.5	-1862.2	12.5
ResultsAAA-Total_run_11_f	11	1	-1868.5	-1862.3	12.4
ResultsAAA-Total_run_18_f	18	1	-1868.5	-1862.2	12.5
ResultsAAA-Total_run_14_f	14	1	-1868.4	-1862.2	12.3
ResultsAAA-Total_run_16_f	16	1	-1868.5	-1862.3	12.4
ResultsAAA-Total_run_17_f	17	1	-1868.5	-1862.3	12.5
ResultsAAA-Total_run_5_f	5	1	-1868.3	-1862.2	12.2
ResultsAAA-Total_run_28_f	28	2	-894.6	-871.0	47.2

ResultsAAA-Total_run_38_f	38	2	-894.9	-871.7	46.4
ResultsAAA-Total_run_39_f	39	2	-892.9	-871.2	43.5
ResultsAAA-Total_run_23_f	23	2	-888.1	-869.8	36.7
ResultsAAA-Total_run_29_f	29	2	-896.8	-870.7	52.2
ResultsAAA-Total_run_34_f	34	2	-894.4	-870.0	48.8
ResultsAAA-Total_run_32_f	32	2	-896.6	-871.0	51.0
ResultsAAA-Total_run_24_f	24	2	-892.2	-869.5	45.3
ResultsAAA-Total_run_22_f	22	2	-888.4	-869.9	37.1
ResultsAAA-Total_run_36_f	36	2	-896.5	-871.5	50.0
ResultsAAA-Total_run_37_f	37	2	-896.0	-871.2	49.6
ResultsAAA-Total_run_26_f	26	2	-898.0	-871.7	52.7
ResultsAAA-Total_run_40_f	40	2	-896.1	-871.2	49.7
ResultsAAA-Total_run_31_f	31	2	-887.8	-869.6	36.4
ResultsAAA-Total_run_33_f	33	2	-889.4	-870.4	38.0
ResultsAAA-Total_run_21_f	21	2	-895.2	-871.1	48.1
ResultsAAA-Total_run_30_f	30	2	-891.9	-871.2	41.5
ResultsAAA-Total_run_35_f	35	2	-889.2	-869.8	38.8
ResultsAAA-Total_run_25_f	25	2	-890.5	-870.4	40.1
ResultsAAA-Total_run_27_f	27	2	-893.9	-870.8	46.1
ResultsAAA-Total_run_54_f	54	3	-804.3	-760.7	87.0
ResultsAAA-Total_run_46_f	46	3	-618.2	-566.8	102.8
ResultsAAA-Total_run_58_f	58	3	-765.5	-734.3	62.3
ResultsAAA-Total_run_49_f	49	3	-761.8	-732.7	58.3
ResultsAAA-Total_run_47_f	47	3	-594.4	-562.9	62.9
ResultsAAA-Total_run_43_f	43	3	-762.5	-734.4	56.2
ResultsAAA-Total_run_52_f	52	3	-618.1	-565.0	106.2
ResultsAAA-Total_run_45_f	45	3	-609.0	-564.9	88.2
ResultsAAA-Total_run_42_f	42	3	-621.2	-569.8	102.9
ResultsAAA-Total_run_57_f	57	3	-612.0	-568.0	88.0
ResultsAAA-Total_run_59_f	59	3	-619.8	-569.1	101.4
ResultsAAA-Total_run_41_f	41	3	-619.0	-567.1	103.7
ResultsAAA-Total_run_56_f	56	3	-763.1	-735.0	56.3
ResultsAAA-Total_run_50_f	50	3	-617.3	-565.7	103.2
ResultsAAA-Total_run_60_f	60	3	-606.2	-565.4	81.6
ResultsAAA-Total_run_53_f	53	3	-606.9	-565.5	82.7
ResultsAAA-Total_run_44_f	44	3	-613.8	-566.3	95.0
ResultsAAA-Total_run_48_f	48	3	-613.8	-567.1	93.4
ResultsAAA-Total_run_51_f	51	3	-744.8	-716.7	56.3
ResultsAAA-Total_run_55_f	55	3	-844.3	-740.4	207.7
ResultsAAA-Total_run_80_f	80	4	-686.7	-648.9	75.6
ResultsAAA-Total_run_66_f	66	4	-594.7	-442.8	303.7
ResultsAAA-Total_run_79_f	79	4	-516.4	-457.7	117.4
ResultsAAA-Total_run_74_f	74	4	-522.2	-457.8	128.8
ResultsAAA-Total_run_68_f	68	4	-519.9	-458.2	123.4
ResultsAAA-Total_run_72_f	72	4	-475.7	-431.6	88.2
ResultsAAA-Total_run_63_f	63	4	-471.4	-415.3	112.2
ResultsAAA-Total_run_71_f	71	4	-927.9	-492.9	869.9
ResultsAAA-Total_run_65_f	65	4	-472.6	-428.7	87.9
ResultsAAA-Total_run_76_f	76	4	-687.2	-649.2	76.0
ResultsAAA-Total_run_73_f	73	4	-478.2	-431.2	93.9

ResultsAAA-Total_run_67_f	67	4	-553.1	-522.1	62.0
ResultsAAA-Total_run_77_f	77	4	-478.6	-431.2	94.8
ResultsAAA-Total_run_70_f	70	4	-525.3	-458.0	134.6
ResultsAAA-Total_run_64_f	64	4	-1028.3	-493.0	1070.6
ResultsAAA-Total_run_61_f	61	4	-517.7	-458.4	118.6
ResultsAAA-Total_run_62_f	62	4	-469.9	-430.2	79.5
ResultsAAA-Total_run_75_f	75	4	-480.6	-432.0	97.2
ResultsAAA-Total_run_78_f	78	4	-526.1	-459.5	133.2
ResultsAAA-Total_run_69_f	69	4	-515.7	-461.3	108.9
ResultsAAA-Total_run_87_f	87	5	-406.2	-350.2	112.0
ResultsAAA-Total_run_99_f	99	5	-410.2	-371.8	76.7
ResultsAAA-Total_run_88_f	88	5	-655.0	-451.8	406.5
ResultsAAA-Total_run_98_f	98	5	-475.1	-414.2	121.8
ResultsAAA-Total_run_82_f	82	5	-405.6	-353.1	105.2
ResultsAAA-Total_run_100_f	100	5	-451.6	-391.8	119.5
ResultsAAA-Total_run_94_f	94	5	-424.9	-388.1	73.5
ResultsAAA-Total_run_96_f	96	5	-487.0	-376.3	221.4
ResultsAAA-Total_run_91_f	91	5	-425.7	-389.0	73.4
ResultsAAA-Total_run_84_f	84	5	-478.1	-414.8	126.5
ResultsAAA-Total_run_85_f	85	5	-632.5	-586.1	92.9
ResultsAAA-Total_run_92_f	92	5	-465.4	-415.5	99.9
ResultsAAA-Total_run_86_f	86	5	-398.3	-347.1	102.5
ResultsAAA-Total_run_89_f	89	5	-424.8	-387.9	73.9
ResultsAAA-Total_run_97_f	97	5	-432.3	-388.6	87.4
ResultsAAA-Total_run_95_f	95	5	-557.6	-419.3	276.7
ResultsAAA-Total_run_90_f	90	5	-505.3	-395.2	220.2
ResultsAAA-Total_run_83_f	83	5	-418.3	-372.4	91.7
ResultsAAA-Total_run_81_f	81	5	-427.9	-388.6	78.7
ResultsAAA-Total_run_93_f	93	5	-468.7	-349.0	239.5
ResultsAAA-Total_run_120_f	120	6	-431.8	-392.5	78.7
ResultsAAA-Total_run_108_f	108	6	-414.3	-306.1	216.6
ResultsAAA-Total_run_112_f	112	6	-350.7	-303.4	94.6
ResultsAAA-Total_run_114_f	114	6	-478.9	-417.8	122.1
ResultsAAA-Total_run_115_f	115	6	-471.9	-418.8	106.1
ResultsAAA-Total_run_107_f	107	6	-418.4	-375.6	85.6
ResultsAAA-Total_run_103_f	103	6	-419.3	-353.4	131.8
ResultsAAA-Total_run_117_f	117	6	-343.0	-287.9	110.0
ResultsAAA-Total_run_106_f	106	6	-428.4	-392.6	71.6
ResultsAAA-Total_run_119_f	119	6	-477.5	-418.4	118.2
ResultsAAA-Total_run_111_f	111	6	-593.9	-322.2	543.5
ResultsAAA-Total_run_113_f	113	6	-344.4	-286.5	115.7
ResultsAAA-Total_run_118_f	118	6	-477.1	-417.6	119.0
ResultsAAA-Total_run_101_f	101	6	-415.7	-376.1	79.1
ResultsAAA-Total_run_104_f	104	6	-354.5	-304.8	99.3
ResultsAAA-Total_run_102_f	102	6	-432.6	-392.5	80.3
ResultsAAA-Total_run_109_f	109	6	-431.3	-393.3	75.9
ResultsAAA-Total_run_110_f	110	6	-417.7	-377.0	81.4
ResultsAAA-Total_run_105_f	105	6	-475.1	-416.9	116.4
ResultsAAA-Total_run_116_f	116	6	-417.2	-376.0	82.4
ResultsAAA-Total_run_122_f	122	7	-300.4	-243.2	114.3

ResultsAAA-Total_run_124_f	124	7	-369.4	-313.8	111.2
ResultsAAA-Total_run_136_f	136	7	-477.2	-420.9	112.7
ResultsAAA-Total_run_123_f	123	7	-426.1	-381.2	89.7
ResultsAAA-Total_run_127_f	127	7	-358.2	-310.4	95.7
ResultsAAA-Total_run_137_f	137	7	-353.9	-310.1	87.5
ResultsAAA-Total_run_135_f	135	7	-358.6	-309.8	97.6
ResultsAAA-Total_run_138_f	138	7	-293.8	-240.0	107.7
ResultsAAA-Total_run_139_f	139	7	-367.1	-309.6	115.0
ResultsAAA-Total_run_128_f	128	7	-300.3	-240.9	118.8
ResultsAAA-Total_run_130_f	130	7	-353.8	-257.0	193.6
ResultsAAA-Total_run_131_f	131	7	-443.9	-399.6	88.6
ResultsAAA-Total_run_126_f	126	7	-365.1	-316.7	96.7
ResultsAAA-Total_run_140_f	140	7	-359.1	-309.8	98.6
ResultsAAA-Total_run_133_f	133	7	-365.2	-311.6	107.3
ResultsAAA-Total_run_129_f	129	7	-350.5	-288.8	123.4
ResultsAAA-Total_run_125_f	125	7	-383.6	-331.7	103.8
ResultsAAA-Total_run_132_f	132	7	-368.3	-310.0	116.6
ResultsAAA-Total_run_134_f	134	7	-359.9	-310.9	98.0
ResultsAAA-Total_run_121_f	121	7	-465.9	-279.5	372.6
ResultsAAA-Total_run_145_f	145	8	-514.1	-238.7	550.7
ResultsAAA-Total_run_155_f	155	8	-367.7	-314.3	106.8
ResultsAAA-Total_run_144_f	144	8	-375.3	-313.5	123.6
ResultsAAA-Total_run_149_f	149	8	-302.3	-247.3	110.0
ResultsAAA-Total_run_148_f	148	8	-331.4	-267.6	127.6
ResultsAAA-Total_run_143_f	143	8	-378.2	-321.4	113.5
ResultsAAA-Total_run_151_f	151	8	-329.4	-267.4	123.9
ResultsAAA-Total_run_154_f	154	8	-322.6	-253.2	138.7
ResultsAAA-Total_run_158_f	158	8	-274.9	-210.8	128.2
ResultsAAA-Total_run_142_f	142	8	-361.8	-311.9	99.9
ResultsAAA-Total_run_141_f	141	8	-300.4	-246.9	107.0
ResultsAAA-Total_run_157_f	157	8	-292.5	-245.6	93.8
ResultsAAA-Total_run_146_f	146	8	-366.6	-314.1	105.1
ResultsAAA-Total_run_160_f	160	8	-308.2	-254.5	107.4
ResultsAAA-Total_run_150_f	150	8	-309.1	-247.1	124.0
ResultsAAA-Total_run_147_f	147	8	-299.8	-247.0	105.6
ResultsAAA-Total_run_159_f	159	8	-380.8	-324.0	113.6
ResultsAAA-Total_run_156_f	156	8	-360.7	-311.8	97.9
ResultsAAA-Total_run_153_f	153	8	-373.7	-320.1	107.3
ResultsAAA-Total_run_152_f	152	8	-476.3	-425.9	100.8
ResultsAAA-Total_run_171_f	171	9	-562.3	-237.0	650.5
ResultsAAA-Total_run_173_f	173	9	-310.4	-229.9	161.0
ResultsAAA-Total_run_170_f	170	9	-302.0	-215.7	172.6
ResultsAAA-Total_run_163_f	163	9	-315.2	-253.5	123.5
ResultsAAA-Total_run_179_f	179	9	-309.9	-252.2	115.3
ResultsAAA-Total_run_166_f	166	9	-309.9	-231.0	157.9
ResultsAAA-Total_run_161_f	161	9	-380.5	-321.0	119.1
ResultsAAA-Total_run_162_f	162	9	-387.8	-326.1	123.5
ResultsAAA-Total_run_174_f	174	9	-317.4	-246.0	142.9
ResultsAAA-Total_run_176_f	176	9	-279.6	-215.1	129.1
ResultsAAA-Total_run_175_f	175	9	-380.3	-320.9	119.0

ResultsAAA-Total_run_164_f	164	9	-272.7	-211.1	123.2
ResultsAAA-Total_run_169_f	169	9	-379.2	-324.9	108.6
ResultsAAA-Total_run_180_f	180	9	-376.9	-269.1	215.7
ResultsAAA-Total_run_172_f	172	9	-373.3	-318.7	109.1
ResultsAAA-Total_run_167_f	167	9	-311.4	-253.9	115.1
ResultsAAA-Total_run_168_f	168	9	-319.5	-254.2	130.5
ResultsAAA-Total_run_178_f	178	9	-378.1	-323.2	109.8
ResultsAAA-Total_run_165_f	165	9	-339.6	-274.7	129.6
ResultsAAA-Total_run_177_f	177	9	-379.1	-321.9	114.5
ResultsAAA-Total_run_187_f	187	10	-282.3	-220.0	124.6
ResultsAAA-Total_run_184_f	184	10	-320.6	-260.9	119.4
ResultsAAA-Total_run_197_f	197	10	-314.0	-258.0	112.0
ResultsAAA-Total_run_181_f	181	10	-424.8	-244.9	359.7
ResultsAAA-Total_run_191_f	191	10	-374.6	-281.4	186.4
ResultsAAA-Total_run_196_f	196	10	-351.1	-280.0	142.3
ResultsAAA-Total_run_189_f	189	10	-291.9	-218.0	147.7
ResultsAAA-Total_run_199_f	199	10	-254.0	-189.3	129.5
ResultsAAA-Total_run_185_f	185	10	-385.3	-324.8	121.0
ResultsAAA-Total_run_195_f	195	10	-327.4	-265.3	124.2
ResultsAAA-Total_run_190_f	190	10	-325.2	-265.4	119.6
ResultsAAA-Total_run_200_f	200	10	-410.1	-269.8	280.6
ResultsAAA-Total_run_188_f	188	10	-318.9	-256.5	124.8
ResultsAAA-Total_run_193_f	193	10	-325.2	-265.4	119.6
ResultsAAA-Total_run_186_f	186	10	-334.3	-268.3	132.2
ResultsAAA-Total_run_198_f	198	10	-393.1	-267.3	251.7
ResultsAAA-Total_run_194_f	194	10	-369.3	-289.4	159.7
ResultsAAA-Total_run_182_f	182	10	-283.5	-215.2	136.7
ResultsAAA-Total_run_183_f	183	10	-363.8	-301.7	124.2
ResultsAAA-Total_run_192_f	192	10	-286.0	-218.9	134.3

(C) Inferred ancestry of individuals based on 212 total barley accessions.

**Serial number**	**Accession number this paper**	**Inferred clusters**		**Serial number**	**Accession number this paper**	**Inferred clusters**	
		Q1	Q2			Q1	Q2
1	HS1	0.997	0.003	107	HS98	0.005	0.995
2	HS10	0.997	0.003	108	HS99	0.006	0.994
3	HS100	0.005	0.995	109	hzd067	0.006	0.994
4	HS101	0.007	0.993	110	hzd068	0.009	0.991
5	HS102	0.007	0.993	111	hzd069	0.005	0.995
6	HS103	0.997	0.003	112	hzd071	0.006	0.994
7	HS104	0.005	0.995	113	hzd079	0.006	0.994
8	HS105	0.006	0.994	114	hzd080	0.975	0.025
9	HS106	0.005	0.995	115	hzd081	0.005	0.995
10	HS107	0.996	0.004	116	hzd082	0.005	0.995
11	HS108	0.006	0.994	117	hzd083	0.006	0.994
12	HS109	0.006	0.994	118	hzd084	0.006	0.994
13	HS11	0.997	0.003	119	hzd085	0.996	0.004

14	HS110	0.006	0.994	120	hzd086	0.007	0.993
15	HS111	0.006	0.994	121	hzd087	0.006	0.994
16	HS112	0.005	0.995	122	hzd098	0.004	0.996
17	HS113	0.006	0.994	123	hzd099	0.005	0.995
18	HS114	0.006	0.994	124	hzd140	0.997	0.003
19	HS115	0.006	0.994	125	hzd141	0.006	0.994
20	HS116	0.006	0.994	126	hzd142	0.006	0.994
21	HS117	0.005	0.995	127	hzd143	0.007	0.993
22	HS118	0.006	0.994	128	hzd144	0.006	0.994
23	HS119	0.006	0.994	129	hzd145	0.996	0.004
24	HS12	0.996	0.004	130	hzd146	0.004	0.996
25	HS13	0.997	0.003	131	hzd147	0.006	0.994
26	HS14	0.997	0.003	132	hzd148	0.005	0.995
27	HS18	0.996	0.004	133	hzd149	0.005	0.995
28	HS19	0.996	0.004	134	hzd175	0.005	0.995
29	HS2	0.997	0.003	135	hzd189	0.006	0.994
30	HS20	0.997	0.003	136	hzd190	0.006	0.994
31	HS21	0.996	0.004	137	hzd191	0.004	0.996
32	HS22	0.997	0.003	138	hzd192	0.005	0.995
33	HS23	0.997	0.003	139	hzd193	0.974	0.026
34	HS25	0.998	0.002	140	hzd194	0.998	0.002
35	HS26	0.997	0.003	141	hzd195	0.005	0.995
36	HS28	0.974	0.026	142	hzd200	0.006	0.994
37	HS29	0.006	0.994	143	hzd201	0.005	0.995
38	HS3	0.996	0.004	144	hzd202	0.998	0.002
39	HS30	0.998	0.002	145	hzd206	0.005	0.995
40	HS31	0.998	0.002	146	hzd208	0.005	0.995
41	HS32	0.997	0.003	147	hzd209	0.006	0.994
42	HS33	0.881	0.119	148	hzd210	0.006	0.994
43	HS34	0.953	0.047	149	hzd213	0.005	0.995
44	HS35	0.976	0.024	150	hzd214	0.009	0.991
45	HS37	0.882	0.118	151	hzd215	0.997	0.003
46	HS38	0.997	0.003	152	hzd216	0.996	0.004
47	HS39	0.996	0.004	153	hzd217	0.997	0.003
48	HS4	0.997	0.003	154	hzd242	0.974	0.026
49	HS40	0.997	0.003	155	hzd247	0.006	0.994
50	HS41	0.997	0.003	156	hzd263	0.006	0.994
51	HS42	0.996	0.004	157	hzd265	0.006	0.994
52	HS43	0.997	0.003	158	hzd288	0.008	0.992
53	HS44	0.997	0.003	159	hzd290	0.006	0.994
54	HS45	0.996	0.004	160	hzd294	0.005	0.995
55	HS46	0.996	0.004	161	hzd295	0.006	0.994
56	HS47	0.997	0.003	162	hzd296	0.005	0.995
57	HS48	0.996	0.004	163	hzd297	0.005	0.995
58	HS49	0.009	0.991	164	hzd298	0.005	0.995
59	HS5	0.997	0.003	165	hzd299	0.005	0.995
60	HS50	0.01	0.99	166	hzd310	0.998	0.002
61	HS51	0.009	0.991	167	hzd311	0.006	0.994
62	HS52	0.898	0.102	168	hzd312	0.006	0.994
63	HS53	0.01	0.99	169	hzd313	0.005	0.995
64	HS54	0.949	0.051	170	hzd314	0.005	0.995

65	HS55	0.008	0.992	171	hzd328	0.006	0.994
66	HS56	0.996	0.004	172	hzd329	0.005	0.995
67	HS57	0.008	0.992	173	hzd330	0.005	0.995
68	HS58	0.008	0.992	174	hzd332	0.005	0.995
69	HS59	0.997	0.003	175	hzd333	0.978	0.022
70	HS60	0.997	0.003	176	hzd334	0.005	0.995
71	HS63	0.996	0.004	177	hzd335	0.005	0.995
72	HS64	0.997	0.003	178	hzd336	0.005	0.995
73	HS65	0.995	0.005	179	hzd378	0.006	0.994
74	HS66	0.997	0.003	180	hzd379	0.006	0.994
75	HS67	0.997	0.003	181	hzd380	0.004	0.996
76	HS68	0.996	0.004	182	hzd381	0.006	0.994
77	HS69	0.996	0.004	183	hzd382	0.006	0.994
78	HS7	0.998	0.002	184	hzd406	0.007	0.993
79	HS70	0.996	0.004	185	hzd407	0.005	0.995
80	HS71	0.996	0.004	186	hzd408	0.007	0.993
81	HS73	0.996	0.004	187	hzd409	0.005	0.995
82	HS74	0.996	0.004	188	hzd416	0.006	0.994
83	HS76	0.974	0.026	189	hzd417	0.006	0.994
84	HS77	0.977	0.023	190	hzd418	0.006	0.994
85	HS78	0.996	0.004	191	hzd419	0.006	0.994
86	HS79	0.996	0.004	192	hzd420	0.006	0.994
87	HS8	0.997	0.003	193	hzd424	0.005	0.995
88	HS80	0.009	0.991	194	hzd425	0.006	0.994
89	HS81	0.996	0.004	195	hzd426	0.005	0.995
90	HS82	0.006	0.994	196	hzd427	0.006	0.994
91	HS83	0.006	0.994	197	hzd428	0.005	0.995
92	HS84	0.005	0.995	198	hzd429	0.006	0.994
93	HS85	0.005	0.995	199	hzd430	0.997	0.003
94	HS86	0.006	0.994	200	hzd489	0.006	0.994
95	HS87	0.996	0.004	201	hzd490	0.007	0.993
96	HS88	0.006	0.994	202	hzd491	0.005	0.995
97	HS89	0.007	0.993	203	hzd494	0.006	0.994
98	HS9	0.006	0.994	204	hzd495	0.005	0.995
99	HS90	0.008	0.992	205	hzd533	0.997	0.003
100	HS91	0.005	0.995	206	hzd539	0.005	0.995
101	HS92	0.006	0.994	207	hzd540	0.006	0.994
102	HS93	0.005	0.995	208	hzd541	0.996	0.004
103	HS94	0.005	0.995	209	hzd542	0.996	0.004
104	HS95	0.005	0.995	210	hzd543	0.996	0.004
105	HS96	0.004	0.996	211	hzd558	0.005	0.995
106	HS97	0.005	0.995	212	hzd560	0.005	0.995

Supplementary Table S3: The code, accession number, name and origin of 212 barley used in this study.

Taxon	Code	Accession No./ Name	Origin (Country)
Hordeum spontaneum	HS1	PI212305	Afghanistan
Hordeum spontaneum	HS2	PI212306	Afghanistan

Hordeum spontaneum	HS3	PI219796	Iraq
Hordeum spontaneum	HS4	PI220664	Afghanistan
Hordeum spontaneum	HS5	PI227019	Iran
Hordeum spontaneum	HS7	PI236386	Syria
Hordeum spontaneum	HS8	PI244772	Pakistan
Hordeum spontaneum	HS9	PI244774	Afghanistan
Hordeum spontaneum	HS10	PI244776	Afghanistan
Hordeum spontaneum	HS11	PI244777	Afghanistan
Hordeum spontaneum	HS12	PI245739	Turkey
Hordeum spontaneum	HS13	PI253933	Iraq
Hordeum spontaneum	HS14	PI254894	Iraq
Hordeum spontaneum	HS18	PI284752	Israel
Hordeum spontaneum	HS19	PI293411	Tajikistan
Hordeum spontaneum	HS20	PI293412	Tajikistan
Hordeum spontaneum	HS21	PI296413	Azerbaijan
Hordeum spontaneum	HS22	PI293414	Azerbaijan
Hordeum spontaneum	HS23	PI296792	Israel
Hordeum spontaneum	HS25	PI296862	Israel
Hordeum spontaneum	HS26	PI296878	Israel
Hordeum spontaneum	HS28	PI354948	Israel
Hordeum spontaneum	HS29	PI356061	Ethiopia
Hordeum spontaneum	HS30	PI356209	Ethiopia
Hordeum spontaneum	HS31	PI391100	Israel
Hordeum spontaneum	HS32	PI401368	Iran
Hordeum spontaneum	HS33	PI401371	Iran
Hordeum spontaneum	HS34	PI420911	Jordan
Hordeum spontaneum	HS35	PI420912	Jordan
Hordeum spontaneum	HS37	PI420915	Jordan
Hordeum spontaneum	HS38	PI420916	Jordan
Hordeum spontaneum	HS39	PI420917	Jordan
Hordeum spontaneum	HS40	PI466040	Syria
Hordeum spontaneum	HS41	PI466048	Syria
Hordeum spontaneum	HS42	PI466060	Syria
Hordeum spontaneum	HS43	PI466086	Syria
Hordeum spontaneum	HS44	PI466118	Syria
Hordeum spontaneum	HS45	PI466130	Syria
Hordeum spontaneum	HS46	PI466178	Syria
Hordeum spontaneum	HS47	PI466206	Syria
Hordeum spontaneum	HS48	PI466238	Syria
Hordeum spontaneum	HS49	PI466249	Lebanon
Hordeum spontaneum	HS50	PI466256	Lebanon
Hordeum spontaneum	HS51	PI466264	Lebanon
Hordeum spontaneum	HS52	PI466296	Israel
Hordeum spontaneum	HS53	PI466328	Israel
Hordeum spontaneum	HS54	PI466388	Israel
Hordeum spontaneum	HS55	PI466498	Israel
Hordeum spontaneum	HS56	PI466524	Israel
Hordeum spontaneum	HS57	PI466554	Israel
Hordeum spontaneum	HS58	PI466586	Israel
Hordeum spontaneum	HS59	PI466605	Iran
Hordeum spontaneum	HS60	PI466618	Iran
Hordeum spontaneum	HS63	PI554426	Turkey
Hordeum spontaneum	HS64	PI466632	Iran
Hordeum spontaneum	HS65	PI466699	Iran
Hordeum spontaneum	HS66	PI554428	Turkey
Hordeum spontaneum	HS67	PI559556	Turkey
Hordeum spontaneum	HS68	PI662052	Tajikistan

Hordeum spontaneum	HS69	PI662080	Tajikistan
Hordeum spontaneum	HS70	PI662109	Tajikistan
Hordeum spontaneum	HS71	PI662118	Tajikistan
Hordeum spontaneum	HS73	PI662158	Turkey
Hordeum spontaneum	HS74	PI662170	Turkey
Hordeum spontaneum	HS76	PI662188	Turkey
Hordeum spontaneum	HS77	PI662204	Turkey
Hordeum spontaneum	HS78	PI662214	Turkey
Hordeum spontaneum	HS79	PI662218	Turkey
Hordeum vulgare	HS80		China
Hordeum vulgare	HS81		China
Hordeum vulgare	HS82		China
Hordeum vulgare	HS83		China
Hordeum vulgare	HS84		China
Hordeum vulgare	HS85		China
Hordeum vulgare	HS86		China
Hordeum vulgare	HS87		China
Hordeum vulgare	HS88		China
Hordeum vulgare	HS89		China
Hordeum vulgare	HS90		China
Hordeum vulgare	HS91		China
Hordeum vulgare	HS92		China
Hordeum vulgare	HS93		China
Hordeum vulgare	HS94		China
Hordeum vulgare	HS95		China
Hordeum vulgare	HS96		China
Hordeum vulgare	HS97		China
Hordeum vulgare	HS98		China
Hordeum vulgare	HS99		China
Hordeum spontaneum	HS100		China
Hordeum spontaneum	HS101		China
Hordeum spontaneum	HS102		China
Hordeum spontaneum	HS103		China
Hordeum spontaneum	HS104		China
Hordeum spontaneum	HS105		China
Hordeum spontaneum	HS106		China
Hordeum spontaneum	HS107		China
Hordeum spontaneum	HS108		China
Hordeum spontaneum	HS109		China
Hordeum spontaneum	HS110		China
Hordeum spontaneum	HS111		China
Hordeum spontaneum	HS112		China
Hordeum spontaneum	HS113		China
Hordeum spontaneum	HS114		China
Hordeum spontaneum	HS115		China
Hordeum spontaneum	HS116		China
Hordeum spontaneum	HS117		China
Hordeum spontaneum	HS118		China
Hordeum spontaneum	HS119		China
Hordeum vulgare	hzd067	Beimai no.4	China
Hordeum vulgare	hzd068	Sakaer	China
Hordeum vulgare	hzd069	Beiqing no.16	China
Hordeum vulgare	hzd071	Dongguoguludeng	China
Hordeum vulgare	hzd079	Dalt	Canada
Hordeum vulgare	hzd080	Ho208	Canada
Hordeum vulgare	hzd081	Mpyt	Canada
Hordeum vulgare	hzd082	changmangzidamai	China

Hordeum vulgare	hzd083	Weixiduanmanglvdamai	China
Hordeum vulgare	hzd084	Zili80qingke	China
Hordeum vulgare	hzd085	Huangqingke	China
Hordeum vulgare	hzd086	Qinyangqumang	China
Hordeum vulgare	hzd087	Zhengzhoudamai	China
Hordeum vulgare	hzd098	Arenter13	Australia
Hordeum vulgare	hzd099	NS293	Australia
Hordeum vulgare	hzd140	W84-152	U.S.A
Hordeum vulgare	hzd141	W84-155	U.S.A
Hordeum vulgare	hzd142	W84-156	U.S.A
Hordeum vulgare	hzd143	W84-158	U.S.A
Hordeum vulgare	hzd144	W84-159	U.S.A
Hordeum vulgare	hzd145	W84-160	U.S.A
Hordeum vulgare	hzd146	W84-161	U.S.A
Hordeum vulgare	hzd147	W84-163	U.S.A
Hordeum vulgare	hzd148	W84-164	U.S.A
Hordeum vulgare	hzd149	W84-165	U.S.A
Hordeum vulgare	hzd175	Zhijiangheshangtou	China
Hordeum vulgare	hzd189	Thunk27	Syria
Hordeum vulgare	hzd190	Owinn	Syria
Hordeum vulgare	hzd191	Lignee527	Syria
Hordeum vulgare	hzd192	Martin46	Syria
Hordeum vulgare	hzd193	Atlas46	Syria
Hordeum vulgare	hzd194	DeirAha106	Syria
Hordeum vulgare	hzd195	Clipper/volla	Syria
Hordeum vulgare	hzd200	Grivita	Rumania
Hordeum vulgare	hzd201	Produtiv	Rumania
Hordeum vulgare	hzd202	W12269	Australia
Hordeum vulgare	hzd206	H0830	Egypt
Hordeum vulgare	hzd208	Quibenras	Columbia
Hordeum vulgare	hzd209	Dram	U.K
Hordeum vulgare	hzd210	Lignee640	France
Hordeum vulgare	hzd213	7200	Sweden
Hordeum vulgare	hzd214	Lina	Sweden
Hordeum vulgare	hzd215	Alva	Sweden
Hordeum vulgare	hzd216	Harry	Sweden
Hordeum vulgare	hzd217	Kristina	Sweden
Hordeum vulgare	hzd242	Baodingmidamai	China
Hordeum vulgare	hzd247	Fengtianbaidamai	China
Hordeum vulgare	hzd263	Jiningjianmangdamai	China
Hordeum vulgare	hzd265	Minheliulengbai	China
Hordeum vulgare	hzd288	Arana	Australia
Hordeum vulgare	hzd290	Supi no.1	China
Hordeum vulgare	hzd294	Jian75	China
Hordeum vulgare	hzd295	Zhahuangzhong no.2	Japan
Hordeum vulgare	hzd296	K5	Japan
Hordeum vulgare	hzd297	K7	Japan
Hordeum vulgare	hzd298	K9	Japan
Hordeum vulgare	hzd299	K10	Japan
Hordeum vulgare	hzd310	Hamidamai	China
Hordeum vulgare	hzd311	Hejiangdamai	China
Hordeum vulgare	hzd312	Jinxingdamai	China
Hordeum vulgare	hzd313	Feite210	China
Hordeum vulgare	hzd314	Danmai no.1	China
Hordeum vulgare	hzd328	grand prix	Netherland
Hordeum vulgare	hzd329	Rorin	Netherland
Hordeum vulgare	hzd330	Atem	Netherland

Hordeum vulgare	hzd332	Lauriner	Canada
Hordeum vulgare	hzd333	Kinkora	Canada
Hordeum vulgare	hzd334	Leger	Canada
Hordeum vulgare	hzd335	Atlanta	Canada
Hordeum vulgare	hzd336	Micmac	Canada
Hordeum vulgare	hzd378	Chuan85-4020	China
Hordeum vulgare	hzd379	Fujiertiao	China
Hordeum vulgare	hzd380	Zhenongda no.2	China
Hordeum vulgare	hzd381	Zhenongda no.3	China
Hordeum vulgare	hzd382	Zhehua no.1	China
Hordeum vulgare	hzd406	Luexuan208	China
Hordeum vulgare	hzd407	Kemai no.1	China
Hordeum vulgare	hzd408	Kemai no.2	China
Hordeum vulgare	hzd409	Kemai no.4	China
Hordeum vulgare	hzd416	Nutans244	Russia
Hordeum vulgare	hzd417	Nutans106	Russia
Hordeum vulgare	hzd418	Mockobckun121	Russia
Hordeum vulgare	hzd419	Australia no. 1	Australia
Hordeum vulgare	hzd420	Arupo"s"	Mexico
Hordeum vulgare	hzd424	Bedford	Mexico
Hordeum vulgare	hzd425	Ideal	Mexico
Hordeum vulgare	hzd426	Gloria"s"/come"s"	Mexico
Hordeum vulgare	hzd427	Gloria"s"/come"s"	Mexico
Hordeum vulgare	hzd428	con"s"/collo"s"	Mexico
Hordeum vulgare	hzd429	H. vulgare 1	Turkey
Hordeum vulgare	hzd430	H. vulgare 2	Turkey
Hordeum vulgare	hzd489	Fu813	China
Hordeum vulgare	hzd490	Minmai8808	China
Hordeum vulgare	hzd491	Minmai8813	China
Hordeum vulgare	hzd494	Sandeli no.5	China
Hordeum vulgare	hzd495	Chengcheng no.17	China
Hordeum vulgare	hzd533	Karan15	Mexico
Hordeum vulgare	hzd539	Ai127	Germany
Hordeum vulgare	hzd540	Ai128	Germany
Hordeum vulgare	hzd541	Ai473	Germany
Hordeum vulgare	hzd542	Ai472	Germany
Hordeum vulgare	hzd543	Ai125	Germany
Hordeum vulgare	hzd558	Theresa	France
Hordeum vulgare	hzd560	Farniwait	Hungary
